# Effect of Xiaonang Yusi decoction (消囊育嗣汤) on IVF outcomes in patients with phlegm-dampness type PCOS: a prospective cohort study with supporting metabolomics, network pharmacology, and molecular docking analysis

**DOI:** 10.3389/fmed.2026.1680327

**Published:** 2026-02-10

**Authors:** Xin Hu, Qingmei Jin, Hengbing Li, Jingyan Song, Haining Yuan, Ying Xu, Kailiang Ai, Zizhen Guo, Zhenni Mu, Zhengao Sun

**Affiliations:** 1 The First Clinical Medical College, Shandong University of Traditional Chinese Medicine, Jinan, China; 2 Department of Reproduction and Genetics, The Affiliated Hospital of Shandong University of Traditional Chinese Medicine, Jinan, China

**Keywords:** arachidonic acid, *in vitro* fertilization-embryo transfer, metabolomics, network pharmacology, phlegm-dampness syndrome, polycystic ovary syndrome, Xiaonang Yusi decoction

## Abstract

**Objective:**

To evaluate the clinical efficacy of Xiaonang Yusi decoction (XNYSD, 消囊育嗣汤) in patients with phlegm-dampness type polycystic ovary syndrome (PCOS) undergoing *in vitro* fertilization and embryo transfer (IVF-ET), and to investigate its underlying mechanism by examining the arachidonic acid (AA) metabolic pathway.

**Methods:**

Women undergoing IVF-ET were divided into a treatment group (TCM treatment), a phlegm-dampness PCOS group, and a tubal factor control group (*n =* 32 each); all received a GnRH antagonist protocol. The study evaluated IVF-ET laboratory and clinical outcomes, alongside changes in TCM syndrome scores. To elucidate the therapeutic mechanism, follicular fluid AA metabolites were quantified by targeted metabolomics. These data were integrated with a network pharmacology analysis to link the active ingredients of Xiaonang Yusi decoction, PCOS targets, and differential metabolites. A protein–protein interaction (PPI) network was then constructed (STRING) and analyzed for GO/KEGG enrichment (DAVID) to systematically clarify the treatment’s mode of action.

**Results:**

Clinical findings demonstrate that Traditional Chinese Medicine (TCM) intervention significantly ameliorates phlegm-dampness symptoms in PCOS patients, with marked improvements in embryo implantation rate (52.0% vs. 28.0%), clinical pregnancy rate (67.9% vs. 29.0%), and ongoing pregnancy rate (60.7% vs. 29.0%) (all *p <* 0.05), while showing no significant intergroup differences in early miscarriage rates (*p >* 0.05). Metabolomic profiling revealed significantly elevated levels of four arachidonic acid metabolites, including 15(S)-hydroxyeicosatetraenoic acid (15(S)-HETE), in follicular fluid of PCOS patients compared to controls (*p <* 0.05), with TCM treatment effectively reducing 15(S)-HETE concentrations (*p <* 0.05). Network pharmacological analysis suggests that Xiaonang Yusi Decoction may modulate PCOS pathophysiology through targeting 15(S)-HETE-mediated pathways, acting on core targets including ESR1 and SIRT1, and influencing critical signaling pathways such as cancer-related pathways, lipid metabolism, and PI3K-Akt signaling. Molecular docking results show favourable interactions, indicating the active compound can spontaneously bind and modulate multiple key targets.

**Conclusion:**

Xiaonang Yusi Decoction significantly improves TCM syndrome manifestations and IVF-ET outcomes in phlegm-dampness PCOS patients, enhancing embryo quality and pregnancy rates. The therapeutic effects appear mediated through regulation of arachidonic acid metabolites, particularly 15(S)-HETE, in follicular fluid. Network pharmacology analysis has preliminarily elucidated the underlying mechanisms, providing novel evidence for TCM-based PCOS treatment strategies.

## 1 Introduction

Polycystic ovary syndrome (PCOS) is a prevalent endocrine and metabolic disorder characterized by hyperandrogenemia, ovulatory dysfunction, and polycystic ovarian morphology, affecting approximately 6–10% of women in their reproductive years (1). This condition demonstrates considerable clinical heterogeneity and is frequently associated with metabolic disturbances, particularly insulin resistance and obesity (2). Although ovulation induction agents (such as letrozole) or *in vitro* fertilization-embryo transfer (IVF-ET) can achieve ovulation in PCOS patients, they continue to experience suboptimal assisted reproductive outcomes, with significantly higher rates of early pregnancy loss compared to non-PCOS populations (3). Current evidence indicates that the endocrine and metabolic dysregulation characteristic of PCOS adversely affects multiple aspects of reproductive physiology. These disturbances impair the follicular microenvironment, disrupt granulosa cell-oocyte communication, and contribute to meiotic abnormalities, collectively resulting in diminished oocyte competence and impaired embryonic development. Furthermore, endometrial dysfunction occurring in the context of hormonal imbalance may represent an additional factor contributing to unfavorable pregnancy outcomes (4–6). These interconnected pathophysiological mechanisms underlie the reproductive challenges observed in women with PCOS.

According to traditional Chinese medicine (TCM) theory, the core pathogenesis of PCOS is closely associated with internal obstruction of phlegm-dampness. Clinical studies indicate that approximately 32.27% of PCOS patients present with spleen deficiency and phlegm-dampness syndrome pattern (7), for which the primary treatment principles focus on fortifying the spleen, drying dampness, and resolving phlegm. Recent research has demonstrated that phlegm-resolving and dampness-drying Chinese herbal medicines can ameliorate PCOS-related metabolic and reproductive abnormalities through multiple mechanisms, including regulation of insulin resistance (IR), androgen levels, and the follicular microenvironment (8–10). Investigation of their potential mechanisms has become an important research direction in reproductive medicine.

Previous studies suggest that Xiaonang Yusi Decoction (XNYSD) may improve reproductive dysfunction in PCOS patients by modulating the arachidonic acid (AA) metabolic pathway (11). As a key *ω*-6 polyunsaturated fatty acid (PUFA), AA is metabolized through cyclooxygenase (COX), lipoxygenase (LOX), and cytochrome P450 (CYP450) pathways to generate bioactive compounds such as prostaglandins (PGs) and leukotrienes (LTs), which are extensively involved in reproductive processes including ovulation and embryo implantation (12). Clinical investigations have revealed that AA metabolic abnormalities play a pivotal role in PCOS pathogenesis. Metabolites produced through COX-2 and CYP450 pathways are significantly elevated in the follicular fluid of PCOS patients (13) and show close correlation with lipid metabolism disorders and ovulatory dysfunction (14). Animal studies have further confirmed that AA metabolic disturbances can impair granulosa cell function, leading to hormonal secretion imbalance and enhanced oxidative stress (15, 16). In summary, AA metabolic dysregulation represents a significant factor contributing to the pathophysiological processes of PCOS.

Targeted metabolomics employs high-sensitivity mass spectrometry to precisely quantify specific metabolite profiles in biological samples (e.g., follicular fluid), thereby revealing dynamic alterations in disease-associated metabolic pathways (17). This advanced technology not only identifies characteristic metabolic fingerprints of PCOS but also tracks metabolic reprogramming following Chinese herbal medicine interventions (7, 18). When integrated with network pharmacology, this approach systematically elucidates the multi-component synergistic mechanisms of compound Chinese formulas by constructing multidimensional “herb-target-pathway-disease” interaction networks (19). Such combined strategies provide innovative methodologies for deciphering the modern scientific principles underlying traditional medicine.

The present study investigates women with phlegm-dampness pattern PCOS and compares them with non-PCOS infertile controls. Patients with phlegm-dampness PCOS received XNYSD intervention, with subsequent evaluation of its clinical effects on IVF-ET outcomes. Simultaneously, based on the AA metabolic pathway theory, we employed targeted metabolomics to quantify AA-related metabolites in follicular fluid across different groups, and integrated network pharmacology analysis to explore the therapeutic targets and underlying mechanisms through which the Chinese herbal medicine improves phlegm-dampness pattern PCOS.

## 2 Materials and methods

### 2.1 Materials

#### 2.1.1 Reagents

HPLC-grade acetonitrile and isopropanol were obtained from Fisher Chemical (Thermo Fisher Scientific Inc., Waltham, MA, USA). Formic acid was purchased from Honeywell International Inc. (Charlotte, NC, USA). Methanol (metabolite extraction solvent) was supplied by Fisher Chemical. Metabolite standards and isotope-labeled standards were acquired from Cayman Chemical Company (Ann Arbor, MI, USA). XNYSD was provided by the Affiliated Hospital of Shandong University of Traditional Chinese Medicine (Jinan, China) and authenticated by Dr. Sun Zhengao (Shandong University of Traditional Chinese Medicine).

#### 2.1.2 Preparation of XNYSD

Based on our clinical experience at the Reproductive Center of the Affiliated Hospital of Shandong University of Traditional Chinese Medicine, the formula for XNYSD is as follows: Yinyanghuo (Herba Epimedii Brevicornus, processed) 12 g, Shichangpu (Rhizoma Acori Tatarinowii) 15 g, Zishiying (Fluoritum) 24 g, Lujiaoshuang (Cornu Cervi Degelatinatum) 15 g, Tusizi (Semen Cuscutae Chinensis) 15 g, Banxia (Rhizoma Pinelliae Ternatae) 9 g, Zhishi (Fructus Aurantii Immaturus) 12 g, Cangzhu (Rhizoma Atractylodis Lanceae) 15 g, Danxing (Arisaema cum Bile) 6 g, Gualou (Fructus Trichosanthis Kirilowii) 15 g, Chongweizi (Fructus Leonuri Japonici) 18 g, Zhixiangfu (Rhizoma Cyperi Rotundi, processed) 15 g, Danshen (Radix Salviae Miltiorrhizae) 15 g, Zhigancao (Radix Glycyrrhizae Uralensis, processed) 6 g.

Preparation, Administration and Quality Control: The preparation process involved a two-stage decoction. The herbs were soaked in distilled water for 30 min, followed by decoction for 30 min. The liquid was filtered, and the residual herbs were re-decocted with distilled water for 30 min. The two decoctions were combined, concentrated to a final volume of 200 mL per daily dose, and stored at 4 °C until use. The experimental group additionally received a standardized daily dose of 200 mL of XNYSD (prepared by the Pharmacy Department of the Affiliated Hospital of Shandong University of Traditional Chinese Medicine) starting from day 5 of the preceding menstrual cycle until the trigger day, administered orally twice daily, half an hour after meals in the morning and evening. The control and normal groups received standard treatment without additional interventions.

##### 2.1.2.1 The basis for the combination of XNYSD

This formula is based on classical theory and clinical experience. Fu Qingzhu’s Gynecology links obesity-related infertility to phlegm-dampness, while Suwen states “all dampness and swelling belong to the spleen.” The spleen (acquired foundation) transforms dampness, and the kidney (congenital foundation) governs reproduction. Their dysfunction leads to phlegm-dampness accumulating in the uterus, causing obesity and reduced fertility.

XNYSD addresses this pathogenesis. Yinyanghuo 12 g and Shichangpu 15 g (sovereign) tonify kidney yang and resolve phlegm. Zishiying 24 g, Lujiaoshuang 15 g, and Tusizi 15 g (minister) support kidney yang; Banxia 9 g, Zhishi 12 g, and Cangzhu 15 g resolve dampness. Danxing 6 g and Gualou 15 g (assistant) clear phlegm-heat; Chongweizi 18 g, Xiangfu 15 g, and Danshen 15 g regulate qi and blood. Gancao 6 g (guide) harmonizes the formula. The combination tonifies kidney and spleen, resolves phlegm, and regulates menstruation.

##### 2.1.2.2 Quality control of XNYSD

The XNYSD used in this study was prepared according to a standardized protocol developed by the Pharmacy Department of the Affiliated Hospital of Shandong University of Traditional Chinese Medicine and authenticated by Dr. Sun Zhengao. All raw herbs were sourced from qualified suppliers and confirmed to meet the standards of the Chinese Pharmacopoeia (2020 edition). Each batch of raw herbs underwent quality control including identification, purity testing (e.g., for heavy metals, pesticides, and microbial contamination). The decoction process was precisely controlled for water volume, temperature, and time to ensure reproducibility. The formula remained fixed during the study; only minor, non-principal herb adjustments (<10% of total dosage) were allowed according to TCM syndrome differentiation, and patients requiring major modifications were excluded.

#### 2.1.3 Major instruments and reagents

Ultra-performance liquid chromatography (UPLC) system (1,290 Infinity II, Agilent Technologies, Santa Clara, CA, USA); Triple quadrupole mass spectrometer (6,500 + QTRAP, SCIEX, Framingham, MA, USA); High-speed centrifuge (5,430 R, Eppendorf AG, Hamburg, Germany); HPLC-grade acetonitrile (A955-4, Fisher Chemical, Waltham, MA, USA); Tissue grinding homogenizer (JXFSTPRP-24 L, Shanghai Jingxin Industrial Co., Ltd., China); Analytical balance (AL104, Mettler Toledo, Greifensee, Switzerland); Ultrasonic cleaner (JP-100 T, Shenzhen Jiemeng Cleaning Equipment Co., Ltd., China); UPLC column (ACQUITY UPLC BEH C18, 1.7 μm, 2.1 mm × 50 mm, Waters Corporation, Milford, MA, USA).

### 2.2 Participants

The sample size was calculated using PASS 2023 software with portable embryo number as the primary outcome measure based on previous studies (20), adopting a statistical power (1-*β*) of 0.8 at a significance level (*α*) of 0.05 while accounting for a 20% dropout rate, which yielded a minimum requirement of 27 cases per group (PCOS group, TCM group, and Normal group). The study initially recruited 96 patients, and after excluding dropouts, 90 patients were ultimately included in the final analysis. The TCM group comprised 28 phlegm-dampness PCOS patients receiving XNYSD intervention, while the PCOS group included 31 phlegm-dampness PCOS patients without Chinese herbal medicine treatment. The Normal group consisted of 31 infertility patients undergoing IVF-ET exclusively due to tubal factors. Diagnostic criteria for infertility were based on the 10th edition of Obstetrics and Gynecology (National Health Commission “14th Five-Year Plan” textbook), and PCOS diagnosis followed the 2023 Expert Consensus on PCOS Diagnosis and Treatment Pathways adult diagnostic standards (21).

#### 2.2.1 TCM symptom pattern identification standard

The diagnostic criteria for phlegm-dampness syndrome were established in accordance with the Guiding Principles for Clinical Research of New Traditional Chinese Medicine Drugs and the 14th Five-Year Plan textbook Gynecology of Traditional Chinese Medicine (edited by Xiaoling Feng and Tingting Zhang). The essential diagnostic requirements include: primary manifestations of infertility, delayed menstruation or even amenorrhea, and obesity; secondary manifestations comprising at least two of the following: scanty menstrual flow, dizziness or chest tightness, sticky sensation in the mouth, fatigue in the limbs, profuse and viscous leukorrhea, and loose stools. The tongue presentation should show a pale and swollen tongue body with possible teeth marks and a white, greasy coating, while the pulse typically presents as slippery or deep-slippery. A definitive diagnosis requires the presence of all primary symptoms accompanied by a minimum of two secondary symptoms, supported by the characteristic tongue and pulse findings.

#### 2.2.2 Inclusion criteria

The study enrolled infertile patients meeting all of the following criteria: (a) aged between 22 and 35 years; (b) diagnosed with phlegm-dampness pattern polycystic ovary syndrome (PCOS); (c) undergoing *in vitro* fertilization-embryo transfer (IVF-ET) treatment; (d) no history of steroidal hormone therapy within the preceding 3 months; (e) normal hepatic and renal function without concurrent gynecological or systemic diseases; (f) receiving antagonist protocol for ovarian stimulation; (g) willingness to provide follicular fluid for experimental research; and (h) signed informed consent with commitment to complete all required treatments and follow-up procedures.

#### 2.2.3 Exclusion criteria

Participants were excluded based on any of the following conditions: (a) age <22 years or >35 years; (b) failure to meet the established diagnostic and inclusion criteria; (c) concurrent infertility factors including endometriosis, premature ovarian failure, ovarian resistance syndrome, or hyperprolactinemia; (d) endocrine disorders involving the hypothalamus, pituitary gland, or adrenal glands; (e) uterine malformations, significant organic endometrial pathologies, or history of pelvic tuberculosis; (f) severe cardiovascular, hepatic, renal, or hematopoietic system diseases; (g) psychiatric disorders; (h) recent hormonal therapy within 3 months prior to enrollment; or (i) known hypersensitivity to the investigational medications.

### 2.3 Methods

#### 2.3.1 Clinical protocols

##### 2.3.1.1 Study medications

The clinical protocol incorporated recombinant follicle-stimulating hormone (r-FSH, Gonal-f; Merck Serono SA Aubonne Branch; Approval no. S2013005575; 450 IU/vial) and human menopausal gonadotropin (HMG; Livzon Pharmaceutical Group Inc., Zhuhai; Approval No. H10940097; 75 IU/vial), supplemented with human chorionic gonadotropin (hCG; same manufacturer as HMG; Approval No. H44020674; 2000 IU/vial) for final oocyte maturation. Ovarian suppression was achieved using gonadotropin-releasing hormone antagonist (GnRH-ant, Orgalutran; Vetter Pharma-Fertigung GmbH & Co. KG; Approval No. H20160574; 0.25 mg/0.5 mL/vial). Luteal phase support comprised progesterone injection (Xianju Pharmaceutical Co., Ltd.; Approval No. H33020828; 20 mg/vial), estradiol valerate tablets (Progynova; DELPHARM Lille S. A. S.; Approval No. J20130009; 1 mg/tablet), and dydrogesterone tablets (Duphaston; Abbott Healthcare Products B. V.; Approval No. H20130110; 10 mg/tablet). The experimental group additionally received XNYSD, produced and quality-controlled by the Pharmacy Department of Affiliated Hospital of Shandong University of Traditional Chinese Medicine according to standardized protocols.

##### 2.3.1.2 Controlled ovarian stimulation protocol

All participants underwent the antagonist protocol, with ovarian stimulation initiated on menstrual cycle day 2–3 following baseline assessment of follicular status and serum hormone levels. The starting gonadotropin (Gn) dose (typically 150–300 IU/day) was determined based on patient age, ovarian reserve, and body mass index (BMI). During the stimulation phase, follicular growth was monitored through regular ultrasonography and hormonal assays, with Gn dosage adjustments made every 2–3 days according to clinical response. GnRH antagonist administration commenced when the leading follicle reached 12–14 mm in diameter or serum estradiol (E2) levels exceeded 200 pg./mL, continuing until the trigger day. The timing of antagonist initiation was individualized to prevent premature luteinizing hormone (LH) surges that could compromise oocyte yield. Final oocyte maturation was triggered when at least three dominant follicles attained ≥17 mm diameter. Transvaginal oocyte retrieval was performed 34–36 h post-trigger, with follicular fluid (FF) collected from mature follicles (16–20 mm diameter) for subsequent targeted AA metabolite analysis.

##### 2.3.1.3 Herbal medicine intervention

Prior to ovarian stimulation, both the experimental and control groups received dydrogesterone for menstrual cycle regulation. The experimental group additionally received daily administration of XNYSD starting from day 5 of the preceding menstrual cycle until the trigger day, while the control and normal groups received standard treatment without additional interventions.

##### 2.3.1.4 Outcome measures

The comprehensive evaluation included baseline characteristics encompassing BMI (kg/m^2^), duration and type of infertility, antral follicle count (AFC), and basal serum levels of follicle-stimulating hormone (FSH), LH, E2, and progesterone (P). Traditional Chinese Medicine syndrome scoring was performed to document changes in phlegm-dampness pattern manifestations before and after treatment, including both composite scores and individual symptom evaluations. Ovarian stimulation parameters comprised Gn administration duration and total dosage, along with LH, E2, and P levels on the trigger day, while monitoring for ovarian hyperstimulation syndrome (OHSS) occurrence. Laboratory outcomes incorporated oocyte retrieval numbers, normal fertilization rates, day 3 grade I embryo counts, number of transferable embryos, and number of high-quality blastocysts. Pregnancy outcomes were assessed through multiple indicators including fresh embryo transfer rates, implantation rates, biochemical and clinical pregnancy rates, early miscarriage rates, and ongoing pregnancy rates following the initial embryo transfer procedure.

Normal fertilization rates: The proportion of normally fertilized oocytes among all inseminated or injected oocytes: Normal fertilization rate = (Number of 2PN oocytes / Number of inseminated oocytes) × 100%.

Number of transferable embryos: The number of embryos meeting the criteria for fresh transfer or cryopreservation according to laboratory morphology standards (D3 or blastocyst stage).

Fresh embryo transfer rates: The proportion of patients who underwent fresh embryo transfer in the same ovarian stimulation cycle.

Implantation rate of the first embryo transfer: The proportion of gestational sacs observed on ultrasound per embryo transferred during the first transfer: Implantation rate = (Number of gestational sacs / Number of embryos transferred) × 100%.

Biochemical pregnancy rate after the first embryo transfer is defined as the proportion of patients who show a positive serum *β*-hCG level (typically ≥25 IU/L) approximately 14 days after the first embryo transfer, without ultrasound evidence of a clinical intrauterine pregnancy at subsequent follow-up.

Biochemical pregnancy rate = Number of β-hCG–positive cases after the first transfer / Total number of first-transfer cycles × 100%.

Clinical pregnancy rate after the first embryo transfer: The proportion of patients with an intrauterine gestational sac and fetal heartbeat confirmed by ultrasound following the first embryo transfer.

Early miscarriage rate after the first embryo transfer: The proportion of pregnancy losses occurring before 12 gestational weeks among clinical pregnancies: Early miscarriage rate = (Number of miscarriages <12 weeks / Number of clinical pregnancies) × 100%.

Ongoing pregnancy rate after the first embryo transfer: The proportion of pregnancies that progressed beyond 12 gestational weeks after the first embryo transfer.

##### 2.3.1.5 Statistical analysis

All data analyses were performed using SPSS 26.0 (IBM Corp., Armonk, NY, USA). Continuous variables were initially assessed for normality distribution. For normally distributed data across the three groups, one-way analysis of variance (ANOVA) was employed with results expressed as mean ± standard deviation (𝑥 ± 𝑠), followed by post-hoc multiple comparisons when significant differences were detected. Non-normally distributed data were analyzed using the Kruskal-Wallis H test and presented as median (interquartile range) [M (Q1, Q3)]. Categorical variables were compared among groups using chi-square tests and reported as number (percentage). Within-group comparisons of normally distributed parameters before and after treatment were conducted using paired t-tests, while the Wilcoxon signed-rank test was applied for non-normally distributed parameters. The threshold for statistical significance was set at *p <* 0.05 for all analyses.

#### 2.3.2 Experimental procedures

##### 2.3.2.1 Sample processing protocol

Follicular fluid samples collected on oocyte retrieval day were centrifuged (3,000 × g, 15 min) to separate the supernatant, which was aliquoted into 1.5 mL Eppendorf tubes and stored at −80 °C. For analysis, 100 μL of thawed sample was mixed with 500 μL BHT protein precipitant and 10 μL of internal standard (1 μg/mL), followed by vortexing (20 s) and centrifugation (14,000 rcf, 10 min, 4 °C). A 400 μL aliquot of the resulting supernatant was diluted with 1,000 μL ultrapure water for solid-phase extraction using Oasis HLB 96-well plates. The purification sequence included: (1) plate activation with 2 mL methanol (1 mL × 2), (2) equilibration with 2 mL water (1 mL × 2), (3) sample loading in multiple aliquots, (4) two-step washing with 2 mL Wash Solution A (1 mL × 2) and 2 mL Wash Solution B (1 mL × 2), and (5) dual-phase elution using 1 mL methanol. The combined eluate was concentrated under nitrogen stream and stored at −80 °C until analysis.

##### 2.3.2.2 Chromatographic conditions

Chromatographic separation was performed using an Agilent 1,290 Infinity LC ultra-high performance liquid chromatography (UHPLC) system. Samples were maintained at 4 °C in the autosampler, with the analytical column temperature set at 35 °C. The mobile phase consisted of (A) 0.1% formic acid in water and (B) 0.1% formic acid in acetonitrile, delivered at a flow rate of 400 μL/min with an injection volume of 2 μL. The gradient elution program was optimized as follows: initial conditions of 30% B were maintained for 1 min, followed by a linear increase to 90% B over 8 min (1–9 min), held at 90% B for 2 min (9–11 min), rapidly returned to 20% B in 0.1 min (11–11.1 min), and re-equilibrated at 20% B for 2.9 min (11.1–14 min).

To ensure system stability and reproducibility, quality control (QC) samples were interspersed at regular intervals throughout the sample sequence. Additionally, standard reference mixtures of target analytes were incorporated into the sample queue for retention time calibration and system performance monitoring.

##### 2.3.2.3 Mass spectrometry conditions

Mass spectrometric analysis was conducted using a 5,500 QTRAP hybrid triple quadrupole/linear ion trap mass spectrometer (SCIEX) operated in negative ionization mode. The electrospray ionization (ESI) source parameters were optimized as follows: source temperature 500 °C, ion source gas 1 (GS1) 50 psi, ion source gas 2 (GS2) 50 psi, curtain gas (CUR) 30 psi, and ion spray voltage floating (ISVF) -4500 V. Multiple reaction monitoring (MRM) mode was employed for targeted ion pair detection.

##### 2.3.2.4 Data processing

Chromatographic peak integration and retention time determination were performed using MultiQuant 3.0.2 software (SCIEX). Metabolite identification was achieved by retention time alignment with authentic chemical standards of target compounds, with mass spectral data matched against reference libraries for confirmation.

#### 2.3.3 Network pharmacology analysis

##### 2.3.3.1 Compound screening and target prediction

Bioactive components of the herbal medicine were systematically screened from the Traditional Chinese Medicine Systems Pharmacology Database (TCMSP)^1^ using predefined pharmacokinetic criteria (oral bioavailability ≥30%, drug-likeness ≥0.18). For compounds unavailable in TCMSP, the HERB database^2^ was consulted as a supplementary resource to identify additional constituents and their molecular targets.

##### 2.3.3.2 Disease target acquisition

PCOS-related targets were retrieved from multiple genomic databases including GeneCards,^3^ Online Mendelian Inheritance in Man (OMIM),^4^ and DisGeNET^5^ using “polycystic ovarian syndrome” as the search term. After removing duplicate entries, all gene identifiers were standardized through UniProt^6^ to ensure consistency.

##### 2.3.3.3 Metabolite target mapping

Small molecule structures of metabolites were queried in PubChem,^7^ with subsequent target prediction performed using SwissTargetPrediction^8^ based on chemical similarity. The intersecting targets among herbal compounds, PCOS-related genes, and metabolite-associated proteins were visualized using Venny 2.1.0^9^ to generate a tripartite Venn diagram.

##### 2.3.3.4 Protein interaction network construction

The shared target genes were analyzed for protein–protein interactions (PPI) via STRING database^10^ with species restricted to *Homo sapiens* and interaction score threshold set at 0.4. The resulting network was downloaded in TSV format and imported into Cytoscape 3.10.1 for topological analysis using CentiScaPe 2.2 plugin, where key nodes were identified based on degree, closeness, and betweenness centrality measures.

##### 2.3.3.5 Functional enrichment analysis

Gene Ontology (GO) and Kyoto Encyclopedia of Genes and Genomes (KEGG) pathway analyses were conducted using DAVID 6.8^11^ with official gene symbols as identifiers. The top 10 significantly enriched terms (*p <* 0.05) in biological processes, cellular components, and molecular functions were selected, along with the top 10 KEGG pathways, to elucidate potential therapeutic mechanisms. Visualization was performed using the bioinformatics online platform.^12^

## 3 Results

### 3.1 Clinical trial outcomes

#### 3.1.1 Participant disposition

The study initially enrolled 96 participants, with subsequent exclusions as follows: two patients from the TCM group were excluded due to non-compliance with herbal medication protocol, two voluntarily withdrew from the trial, one PCOS group participant was removed following cycle cancellation, and one Normal group subject was excluded upon post-enrollment discovery of ineligibility. This yielded a final analytical cohort of 90 patients (TCM: *n =* 28; PCOS: *n =* 31; Normal: *n =* 31).

#### 3.1.2 Baseline demographic and clinical characteristics

##### 3.1.2.1 Baseline characteristics of the three patient groups

Comparative analysis revealed no statistically significant differences among the three groups regarding duration of infertility, infertility type, basal E2, or P levels (all *p >* 0.05). While BMI, basal FSH, and AFC showed comparable values between the PCOS and TCM groups (*p >* 0.05), both groups exhibited significantly higher values than the Normal group (*p <* 0.05). Notably, the PCOS group demonstrated elevated basal LH levels compared to both the TCM and Normal groups (*p <* 0.05). Complete statistical results are presented in Table 1.

**Table 1:** Basic data of patients in the three groups [*x–*±s, M(Q1, Q3), No. (%)].

Item	Normal group (*n =* 31)	PCOS group (*n =* 31)	TCM group (*n =* 28)	*P*
BMI (kg/m ^2^)	21.90 (20.10, 22.80)	29.90 (26.40, 33.10)*	28.40 (26.03, 30.08)*	< 0.001
Infertility years (years)	3 (1, 3)	3 (2, 6)	3 (1, 5)	0.203
Type of Infertility				0.522
Primary Infertility	11 (35.5)	8 (25.8)	11 (39.3)	
Secondary Infertility	20 (64.5)	23 (74.2)	17 (60.7)	
Basic FSH (IU/L)	6.42 (5.60, 7.74)	4.92 (4.37, 5.79)*	5.25 (4.27, 6.39)*	< 0.001
Basic LH (mIU/mL)	4.16 (3.45, 6.74)	8.43 (5.79, 10.99)*	4.49 (3.36, 7.35)^#^	< 0.001
Basic E2 (pg/ml)	38.35 (31.97, 47.68)	42.23 (30.67, 49.17)	32.65 (26.13, 40.43)	0.057
Basic P (ng/ml)	0.42 (0.32, 0.71)	0.39 (0.15, 0.56)	0.36 (0.18, 0.68)	0.537
AFC	13 (11, 15)	17 (13, 26)*	17 (13, 25)*	0.010

*Statistically different compared to the Normal group; ^#^Statistically different compared to the PCOS group.

##### 3.1.2.2 Changes in the evidence points of the three groups of patients after XNYSD medication

The TCM evidence points of the XNYSD TCM intervention group (TCM group) were significantly reduced after TCM treatment (*p <* 0.05), and the total points were reduced by 31.42%, which proved that the Eliminating Capsule and Yuzi Soup was able to effectively improve the symptoms of phlegm-dampness of PCOS patients. See Table 2 for details.

**Table 2:** Changes in traditional Chinese medicine syndrome scores before and after treatment in the XNYSD group and before and after conventional treatment in the PCOS group (mean ± SD).

Group	Pre-treatment	Post-treatment	*P*	Decrease in total points (%)
TCM group	15.57 ± 2.348	10.68 ± 2.039	< 0.001	31.42%
PCOS group	15.29 ± 1.950	14.48 ± 2.093	0.235	5.30%
*P*	0.802	< 0.001		

Following administration of XNYSD in the TCM group, patients exhibited significantly lower syndrome scores compared to pre-treatment levels (all *p <* 0.05) for a range of symptoms, including abnormalities in menstrual volume, color, and quality; excessive vaginal discharge; a sticky sensation in the mouth; epigastric and abdominal fullness; lassitude of the limbs; and abnormal bowel, tongue, and pulse conditions. In contrast, scores for obesity, menstrual cycle, and a sensation of heaviness in the head and body did not show a significant change post-treatment (all *p >* 0.05), as detailed in Table 3.

**Table 3:** TCM group: Scores for individual symptoms related to phlegm-dampness pattern before and after treatment PCOS group: scores for individual symptoms related to phlegm-dampness pattern (mean ± SD).

TCM Group	Pre-treatment	Post-treatment	*P*
Obesity	1.57 ± 0.573	1.50 ± 0.577	0.161
Menstrual cycle	2.00 ± 0.720	1.82 ± 0.612	0.057
Abnormalities in menstrual volume, color, and quality	1.39 ± 0.685	0.93 ± 0.716	< 0.001
Excessive vaginal discharge	1.00 ± 0.720	0.75 ± 0.645	0.006
A sticky sensation in the mouth	1.14 ± 0.591	0.86 ± 0.525	0.003
Sensation of heaviness in the head and body	0.79 ± 0.568	0.68 ± 0.548	0.083
Epigastric and abdominal fullness	1.04 ± 0.637	0.82 ± 0.612	0.011
Lassitude of the limbs	0.75 ± 0.645	0.50 ± 0.509	0.006
Bowel movements	2.07 ± 0.766	0.75 ± 0.518	< 0.001
Tongue body	1.86 ± 0.651	1.11 ± 0.685	< 0.001
Tongue coating	1.96 ± 0.744	0.96 ± 0.508	< 0.001
Pulse condition	1.36 ± 0.951	0.86 ± 1.008	0.006
PCOS Group	pre-treatment	post-treatment	*P*
Obesity	1.55 ± 0.580	1.53 ± 0.575	0.214
Menstrual cycle	2.02 ± 0.715	1.84 ± 0.618	0.035
Abnormalities in menstrual volume, color, and quality	1.38 ± 0.690	0.95 ± 0.720	< 0.001
Excessive vaginal discharge	1.02 ± 0.710	0.98 ± 0.690	0.076
A sticky sensation in the mouth	1.15 ± 0.595	1.11 ± 0.570	0.134
Sensation of heaviness in the head and body	0.80 ± 0.570	0.78 ± 0.560	0.083
Epigastric and abdominal fullness	1.05 ± 0.640	0.78 ± 0.560	0.101
Lassitude of the limbs	0.76 ± 0.650	0.72 ± 0.610	0.235
Bowel movements	2.05 ± 0.770	1.95 ± 0.740	0.802
Tongue body	1.85 ± 0.655	1.75 ± 0.680	0.793
Tongue coating	1.95 ± 0.750	1.82 ± 0.710	0.532
Pulse condition	1.38 ± 0.940	1.31 ± 0.980	0.235

#### 3.1.3 Outcomes of controlled ovarian hyperstimulation in the three groups

Compared with the Normal group, patients in the PCOS group required a longer duration of Gn administration and a higher total Gn dosage. Furthermore, both the PCOS and TCM groups exhibited higher serum LH levels on the trigger day and a greater incidence of OHSS (all *p <* 0.05). Although the total Gn dosage was reduced in the TCM group following the herbal intervention, this difference was not statistically significant when compared to the PCOS group (*p >* 0.05). There were no significant differences in trigger-day E2 and P levels among the three groups (all *p >* 0.05). Details are provided in Table 4.

**Table 4:** Parameters of controlled ovarian hyperstimulation in the three groups [mean ± SD; median (Q1, Q3)].

Item	Normal group (*n =* 31)	PCOS group (*n =* 31)	TCM group (*n =* 28)	*p*
Gn duration (days)	8 (8, 9)	9 (9, 10)*	9 (8, 10)	0.009
Total Gn dose (IU)	1700 (1,425, 1950)	2,100 (1825, 2,475)*	1825 (1,681, 2025)	<0.001
Trigger-day LH (mIU/mL)	1.58 (1.16, 2.51)	3.63 (1.85, 5.31)*	2.68 (1.73, 4.81)*	<0.001
Trigger-day E2 (pg/mL)	2493.72 (1776.63, 3150.26)	3000.00 (1968.91, 4992.12)	3230.40 (1913.85, 4508.75)	0.239
Trigger-day P (ng/mL)	1.00 (0.63, 1.19)	1.02 (0.53, 1.50)	0.81 (0.55, 1.50)	0.904
OHSS	7 (16.7)	17 (40.5)*	18 (42.9)*	0.003

*Indicates a statistically significant difference compared with the Normal group; ^#^Indicates a statistically significant difference compared with the PCOS group.

#### 3.1.4 Laboratory outcomes in the three groups

Following the intervention with XNYSD, the TCM group, when compared to the PCOS group, demonstrated a significantly higher number of normally fertilized oocytes, Day 3 Grade I embryos, transferable embryos, and high-quality blastocysts, as well as a higher normal fertilization rate (all *p <* 0.05). The number of oocytes retrieved did not differ significantly between these two groups (*p >* 0.05). For details, see Table 5.

**Table 5:** Laboratory outcomes in the three groups [mean ± SD; median (Q1, Q3)].

Item	Normal group (*n =* 31)	PCOS group (*n =* 31)	TCM group (*n =* 28)	*P*
Number of oocytes retrieved	12 (9, 15)	14 (10, 20)	16 (11, 21)*	0.042
Number of normally fertilized oocytes	9 (6, 11)	8 (5, 12)	12 (8, 16)*^#^	0.038
Normal fertilization rate (%)	75.8 ± 16.4	63.0 ± 21.8*	78.7 ± 15.6^#^	0.004
Number of Day 3 Grade I embryos	1 (0, 3)	1 (0, 2)	2 (1, 3)^#^	0.014
Number of transferable embryos	4 (3, 6)	4 (3, 5)	6 (5, 7)*^#^	0.003
Number of high-quality blastocysts	0 (0, 2)	0 (0, 1)	2 (1, 2)*^#^	0.004

**Indicates a statistically significant difference compared with the Normal group; ^#^Indicates a statistically significant difference compared with the PCOS group.

#### 3.1.5 Pregnancy outcomes in the three groups

Statistically significant differences were observed among the three groups regarding the fresh embryo transfer cycle rate, as well as the implantation, biochemical pregnancy, clinical pregnancy, and ongoing pregnancy rates following the first embryo transfer (all *p <* 0.05). Post-hoc analysis using the Bonferroni correction revealed that the fresh embryo transfer cycle rate was higher in the Normal group than in the PCOS group (54.8% vs. 22.6%, *p <* 0.05) and the TCM group (54.8% vs. 14.3%, *p <* 0.05). Compared to the PCOS group, patients in the Normal group exhibited higher rates of implantation (50.0% vs. 28.0%, *p <* 0.05), biochemical pregnancy (80.0% vs. 48.4%, *p <* 0.05), clinical pregnancy (66.7% vs. 29.0%, *p <* 0.05), and ongoing pregnancy (60.0% vs. 29.0%, *p <* 0.05) after the first embryo transfer. Following the intervention with XNYSD, the TCM group showed significant improvements over the PCOS group in the first-embryo-transfer implantation rate (52.0% vs. 28.0%, *p <* 0.05), clinical pregnancy rate (67.9% vs. 29.0%, *p <* 0.05), and ongoing pregnancy rate (60.7% vs. 29.0%, *p <* 0.05). Although the biochemical pregnancy rate in the TCM group improved compared to the PCOS group, the difference was not statistically significant (*p >* 0.05). There was no statistically significant difference in the early miscarriage rate after the first embryo transfer among the three groups (*p >* 0.05). For details, see Table 6.

**Table 6:** Pregnancy outcomes in the three groups [*n* (%)].

Item	Normal group (*n =* 31)	PCOS group (*n =* 31)	TCM group (*n =* 28)	*P*
Fresh embryo transfer cycle rate (%)	17 (54.8)	7 (22.6)*	4 (14.3)*	0.002
Implantation rate after first ET (%)^a^	21 (50.0)	14 (28.0)*	26 (52.0)^#^	0.029
Biochemical pregnancy rate after first ET (%)^a^	24 (80.0)	15 (48.4)*	20 (71.4)	0.026
Clinical pregnancy rate after first ET (%)^a^	20 (66.7)	9 (29.0)*	19 (67.9)^#^	0.003
Early miscarriage rate after first ET (%)^a^	2 (7.1)	0 (0.0)	2 (6.7)	0.325
Ongoing pregnancy rate after first ET (%)^a^	18 (60.0)	9 (29.0)*	17 (60.7)^#^	0.019

*Indicates a statistically significant difference compared with the Normal group; ^#^Indicates a statistically significant difference compared with the PCOS group; ^a^One patient in the Normal group did not undergo embryo transfer during the follow-up period.

### 3.2 Results of metabolomics analysis

#### 3.2.1 Sample information

Table 7 provides the information for the samples subjected to targeted metabolomics. For this analysis, follicular fluid was collected on the day of oocyte retrieval from 16 randomly selected patients from each group for the detection of AA-related metabolites.

**Table 7:** Sample information.

Group	Group name	Quantity	Sample status
1	TCM	16	Fluid
2	PCOS	16	Fluid
3	Normal	16	Fluid

#### 3.2.2 Quantification of analytes and assessment of data stability

**Figure 1:**
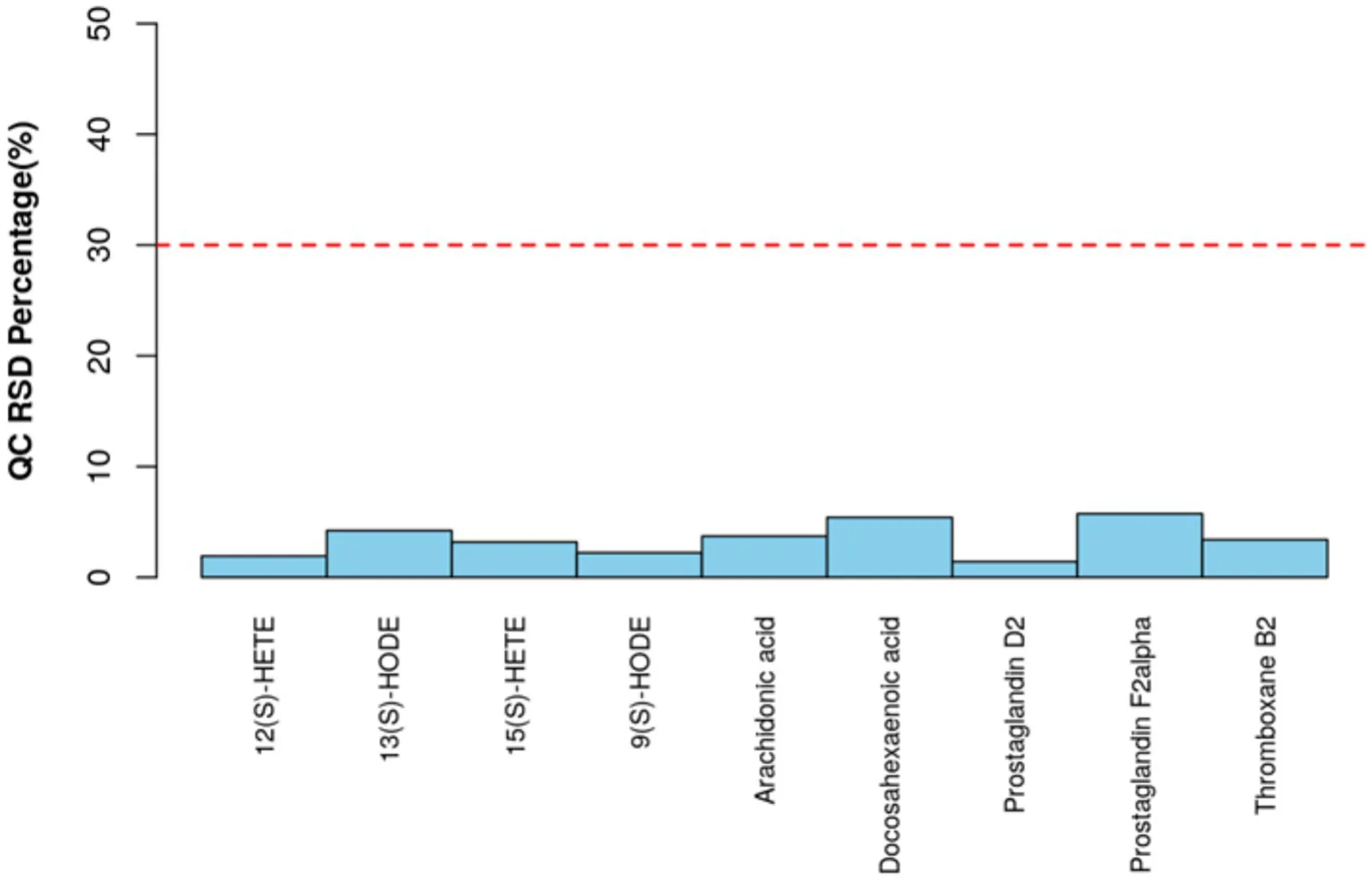
Stability analysis of target analytes based on the relative standard deviation (RSD) in QC samples.

A quality control (QC) sample was prepared by pooling equal aliquots from all individual samples to evaluate the stability and repeatability of the data. The stability of the data for each analyte was assessed based on its relative standard deviation (RSD) in the QC samples, with an RSD of less than 30% considered stable and reliable. As illustrated in Figures 1, a total of nine metabolites were identified in this experiment, all of which met this stability criterion.

#### 3.2.3 Inter- and intragroup variations in metabolites

**Figure 2:**
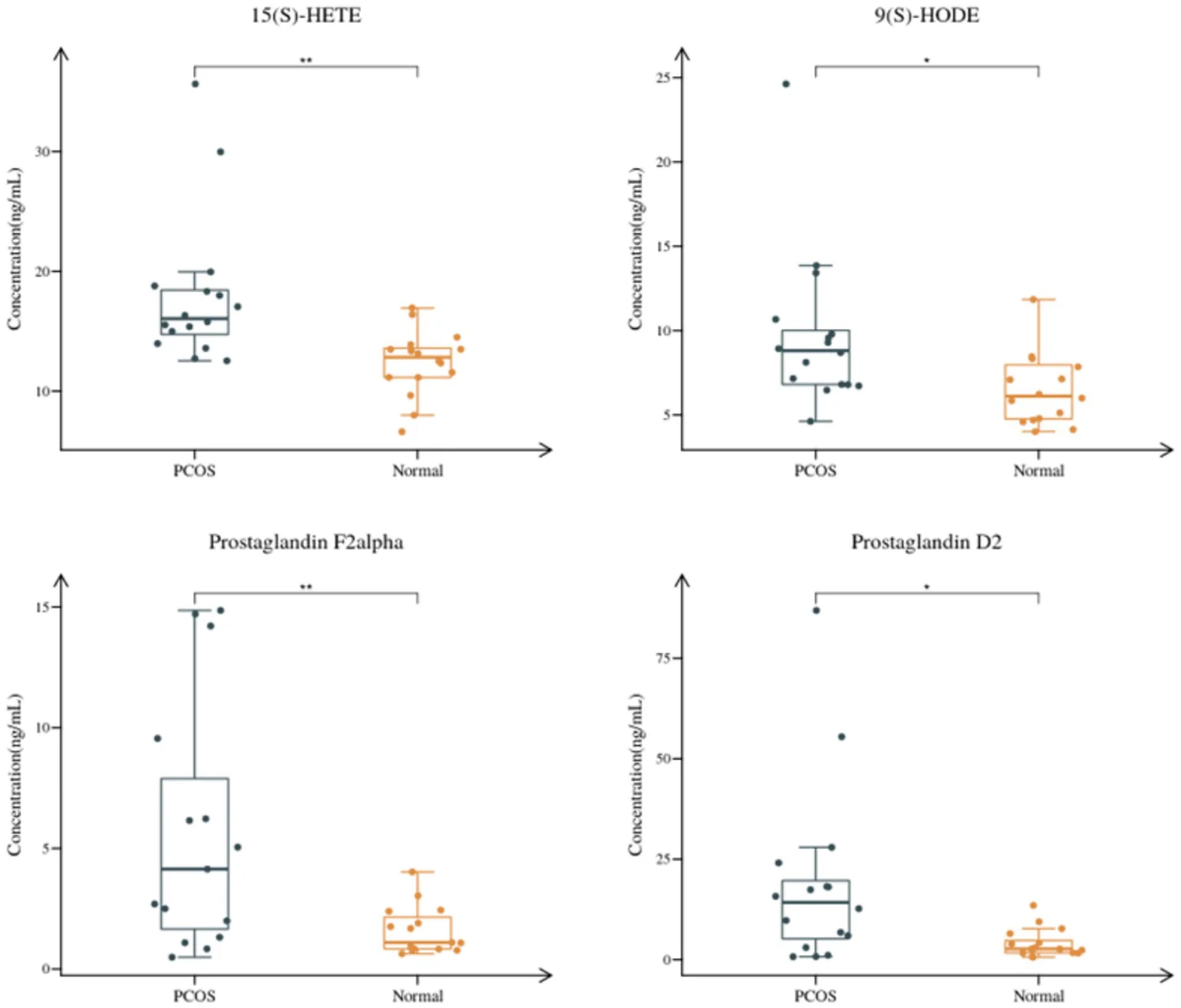
Levels of differential metabolites between the PCOS and normal groups.

**Figure 3:**
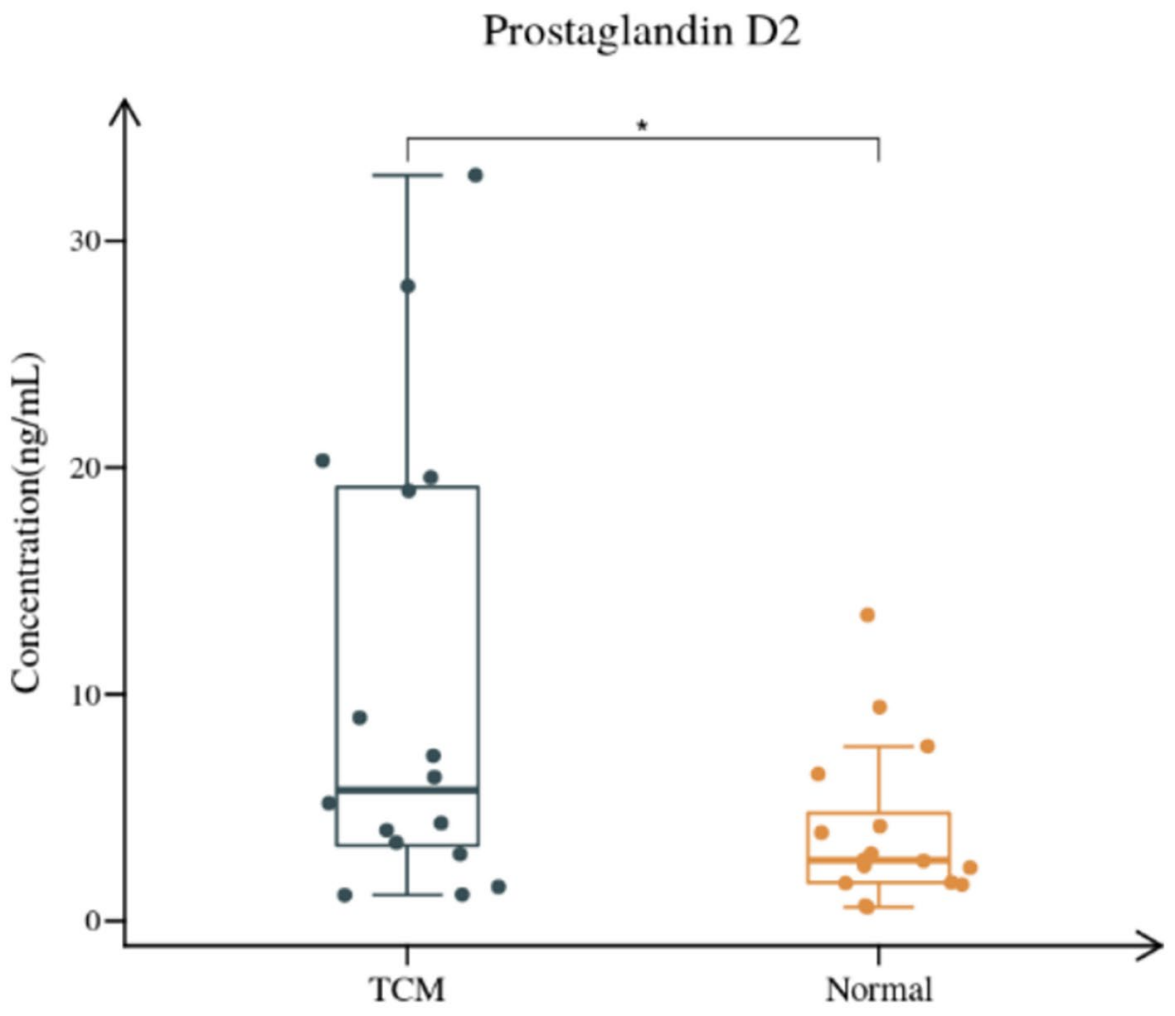
Levels of differential metabolites between the TCM and normal groups.

**Figure 4:**
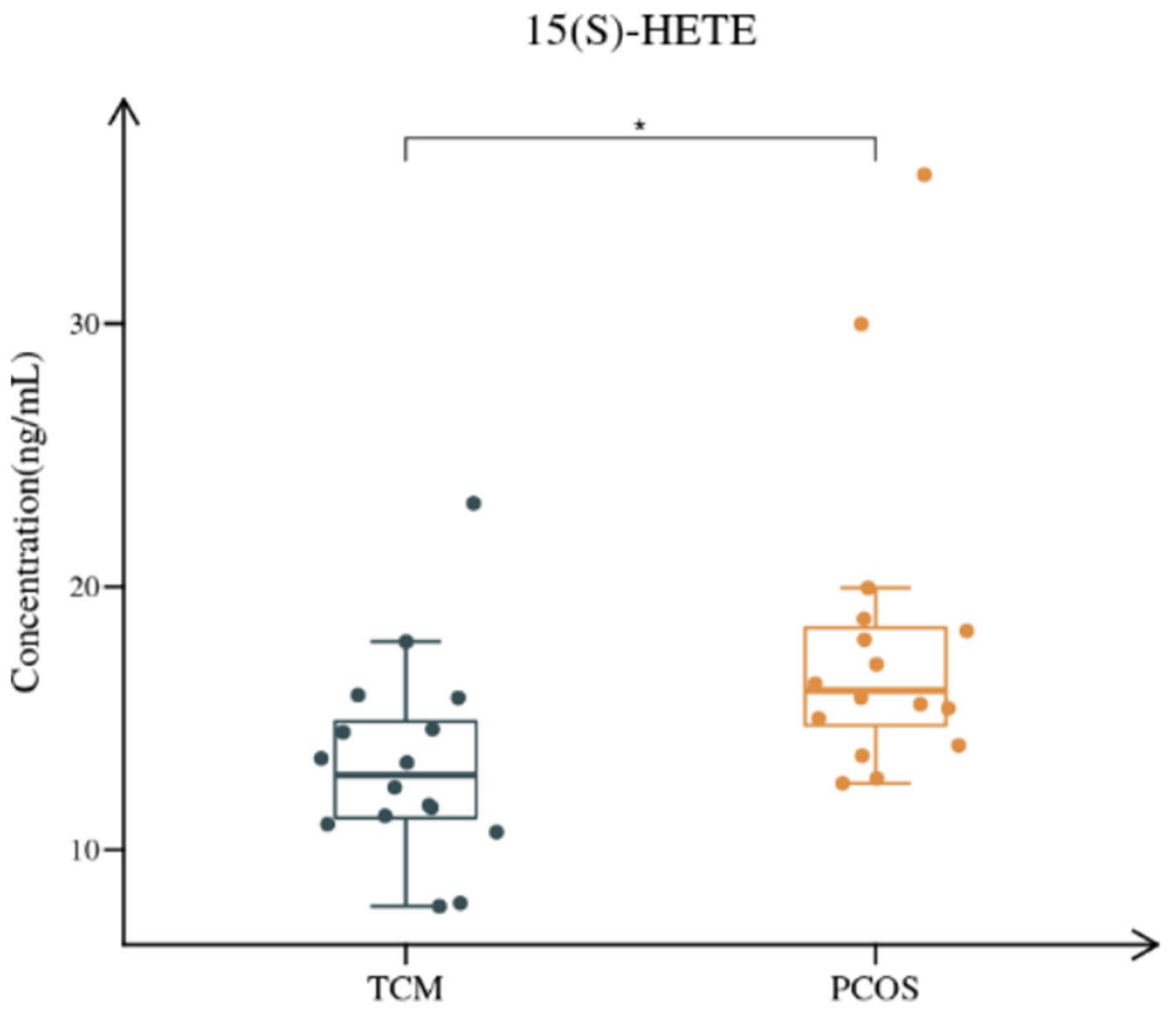
Levels of differential metabolites between the TCM and PCOS groups. The *x*-axis represents the experimental groups, and the *y*-axis indicates the metabolite concentration. *p*-values were determined using either a *t*-test or analysis of variance (ANOVA). The symbols ** and * represent *p <* 0.01 and *p <* 0.05, respectively.

The results of the quantitative mass spectrometry analysis for metabolites in each group are presented as box plots. The data revealed that, compared to the Normal group, the follicular fluid of the PCOS group contained higher levels of 15(S)-HETE, 9(S)-hydroxyoctadecadienoic acid (9(S)-HODE), prostaglandin F2alpha (PGF2α), and prostaglandin D2 (PGD2) (Figure 2). Similarly, the level of PGD2 in the follicular fluid of the TCM group was higher than in the Normal group (Figure 3), while the level of 15(S)-HETE was lower (Figure 4). Following the Chinese medicine intervention, the level of 15(S)-HETE in the follicular fluid of patients with phlegm-dampness type PCOS was significantly reduced.

### 3.3 Results of network pharmacology analysis

#### 3.3.1 Prediction of active ingredients and targets for XNYSD

Active ingredients of the XNYSD were identified by searching the Traditional Chinese Medicine Systems Pharmacology (TCMSP) database. Using the screening criteria of oral bioavailability (OB) ≥ 30% and drug-likeness (DL) ≥ 0.18, and after excluding compounds without predicted targets, a total of 254 active ingredients were collected. This included 88 from Glycyrrhiza uralensis (Gancao), 59 from *Salvia miltiorrhiza* (Danshen), 1 from Amethystum (Zishiying), 12 from *Pinellia ternata* (Banxia), 4 from Atractylodes lancea (Cangzhu), 1 from *Leonurus japonicus* (Chongweizi), 4 from Arisaema cum Bile (Dannnxing), 10 from Trichosanthes kirilowii (Gualou), 2 from Cornu Cervi Degelatinatum (Lujiaoshuang), 4 from Acorus tatarinowii (Shichangpu), 10 from Cuscuta chinensis (Tusizi), 16 from *Cyperus rotundus* (Xiangfu), 23 from Epimedium brevicornu (Yinyanghuo), and 20 from *Citrus aurantium* (Zhishi). The targets corresponding to these ingredients were integrated, and after the removal of duplicates, a total of 365 potential targets for the herbal formula were identified.

#### 3.3.2 Identification of PCOS-associated targets

Disease-related targets for PCOS were compiled by integrating data from the GeneCards, Online Mendelian Inheritance in Man (OMIM), and DisGeNET databases. After removing duplicates, a total of 4,597 PCOS-associated targets were obtained. The gene names for these targets were subsequently standardized using the UniProt database.

#### 3.3.3 Prediction of targets associated with the metabolite 15(S)-HETE

**Figure 5:**
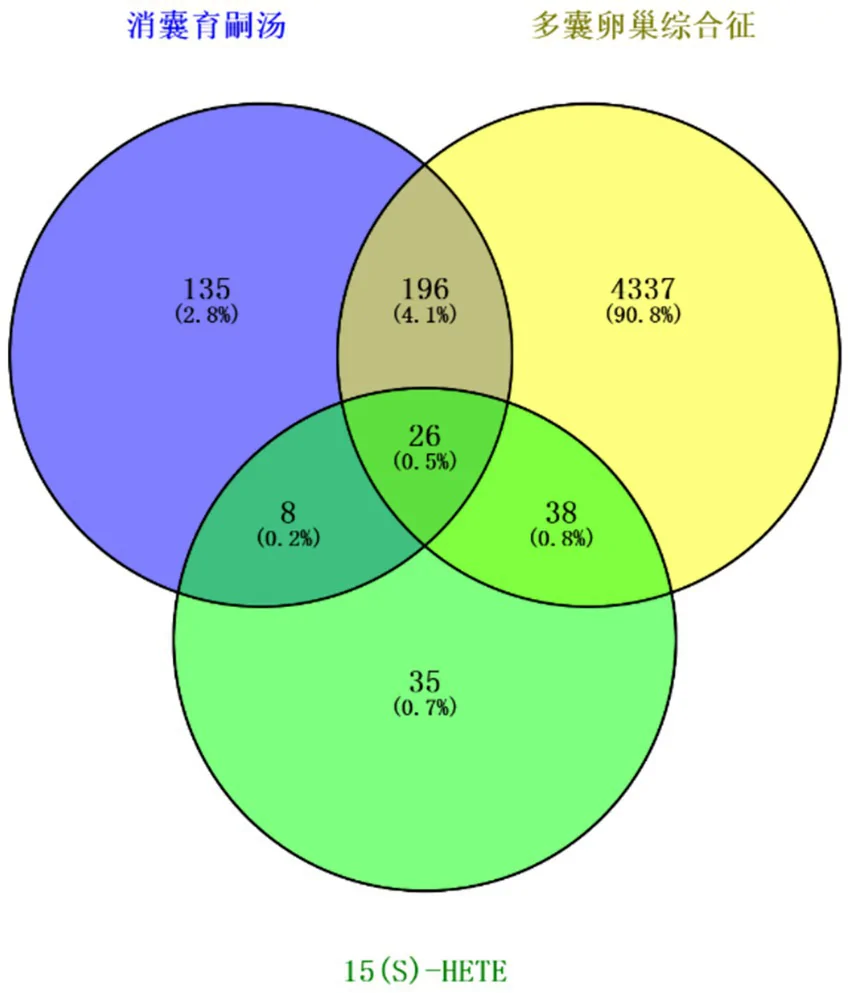
Venn diagram of the intersection targets among the drug, disease, and metabolite.

The chemical structure of 15(S)-HETE was retrieved from the PubChem database. Based on chemical similarity, potential protein targets were predicted using the SwissTargetPrediction platform. After the removal of duplicates, this process yielded 107 targets associated with 15(S)-HETE. A Venn diagram was then constructed to visualize the intersection of the drug targets (from XNYSD), the disease targets (from PCOS), and the metabolite targets (from 15(S)-HETE). The analysis revealed 26 common targets at the intersection, as illustrated in Figure 5.

#### 3.3.4 Protein–protein interaction (PPI) network of intersection targets

**Figure 6:**
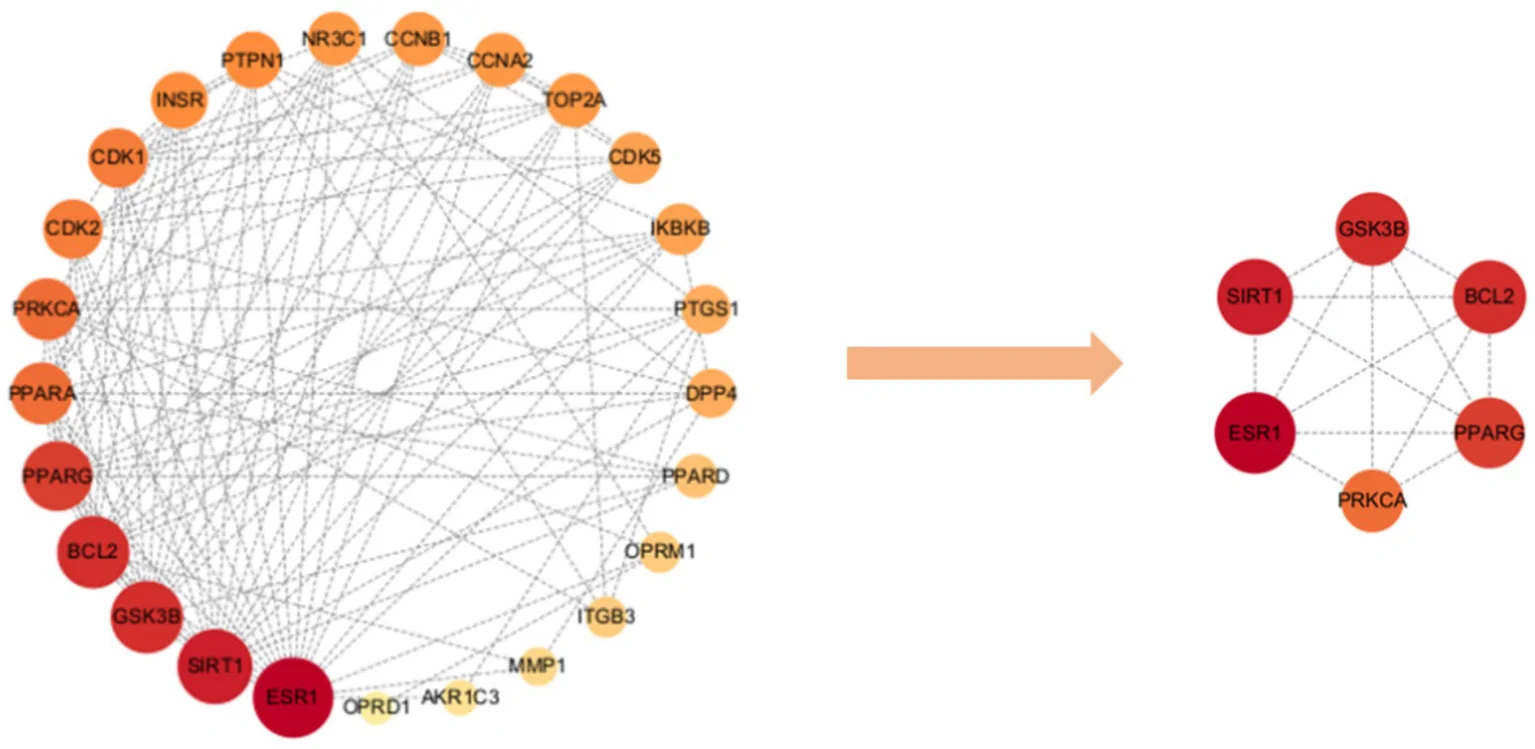
Protein–protein interaction (PPI) network of the intersection targets and identification of hub targets.

The 26 intersection targets were imported into the STRING database to construct a Protein–Protein Interaction (PPI) network. Subsequently, the network was analyzed using Cytoscape software (version 3.8.2) to identify hub targets. Based on the screening criteria of closeness > 0.0251, betweenness > 17.04, and degree > 9.36, a total of six key targets were identified, as illustrated in Figure 6. These targets are: estrogen receptor alpha (ESR1), NAD-dependent deacetylase sirtuin-1 (SIRT1), glycogen synthase kinase-3 beta (GSK3B), apoptosis regulator Bcl-2 (BCL2), peroxisome proliferator-activated receptor gamma (PPARG), and protein kinase C alpha (PRKCA). It is postulated that these core targets play a crucial role in the therapeutic mechanism of XNYSD in the treatment of phlegm-dampness type PCOS.

#### 3.3.5 GO enrichment analysis

**Figure 7:**
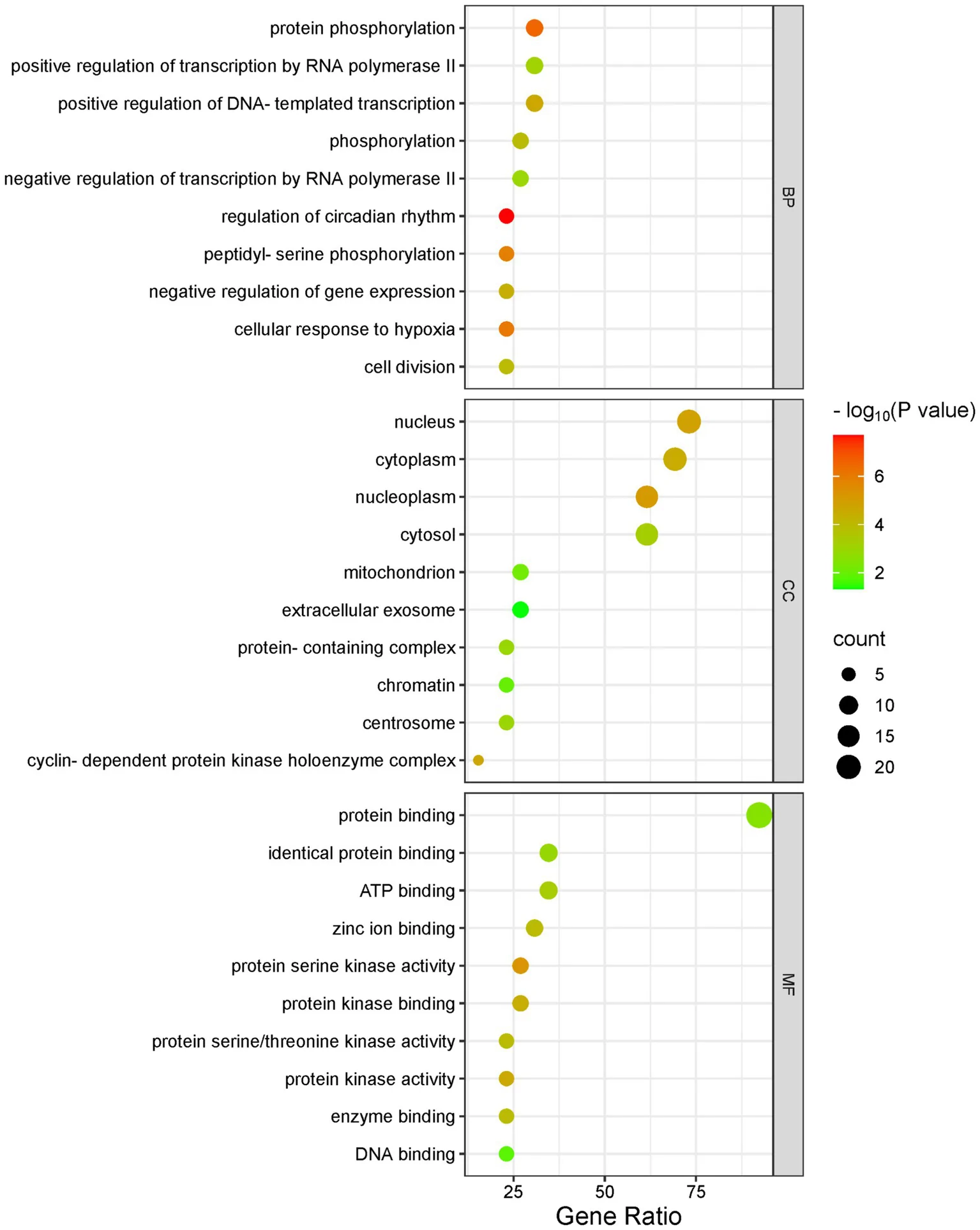
Gene ontology (GO) enrichment analysis of the intersection targets.

Gene Ontology (GO) and Kyoto Encyclopedia of Genes and Genomes (KEGG) pathway enrichment analyses were performed on the intersection genes using the DAVID database, and the results were subsequently visualized. The GO enrichment analysis yielded a total of 203 terms, comprising 133 in Biological Process (BP), 25 in Cellular Component (CC), and 45 in Molecular Function (MF). Using a significance threshold of *p <* 0.05 and ranking by gene ratio, the top 10 most significantly enriched terms for each category are displayed in Figure 7. The top enriched Biological Processes included protein phosphorylation, positive regulation of DNA-templated transcription, positive regulation of transcription by RNA polymerase II, phosphorylation, negative regulation of transcription by RNA polymerase II, regulation of circadian rhythm, cellular response to hypoxia, peptidyl-serine phosphorylation, negative regulation of gene expression, and cell division. For Cellular Component, the most significant terms were nucleus, cytoplasm, nucleoplasm, cytosol, mitochondrion, extracellular exosome, centrosome, protein-containing complex, chromatin, and cyclin-dependent protein kinase holoenzyme complex. The predominant Molecular Functions consisted of protein binding, ATP binding, identical protein binding, zinc ion binding, protein serine kinase activity, protein kinase binding, protein kinase activity, enzyme binding, protein serine/threonine kinase activity, and DNA binding.

#### 3.3.6 KEGG pathway enrichment analysis

**Figure 8:**
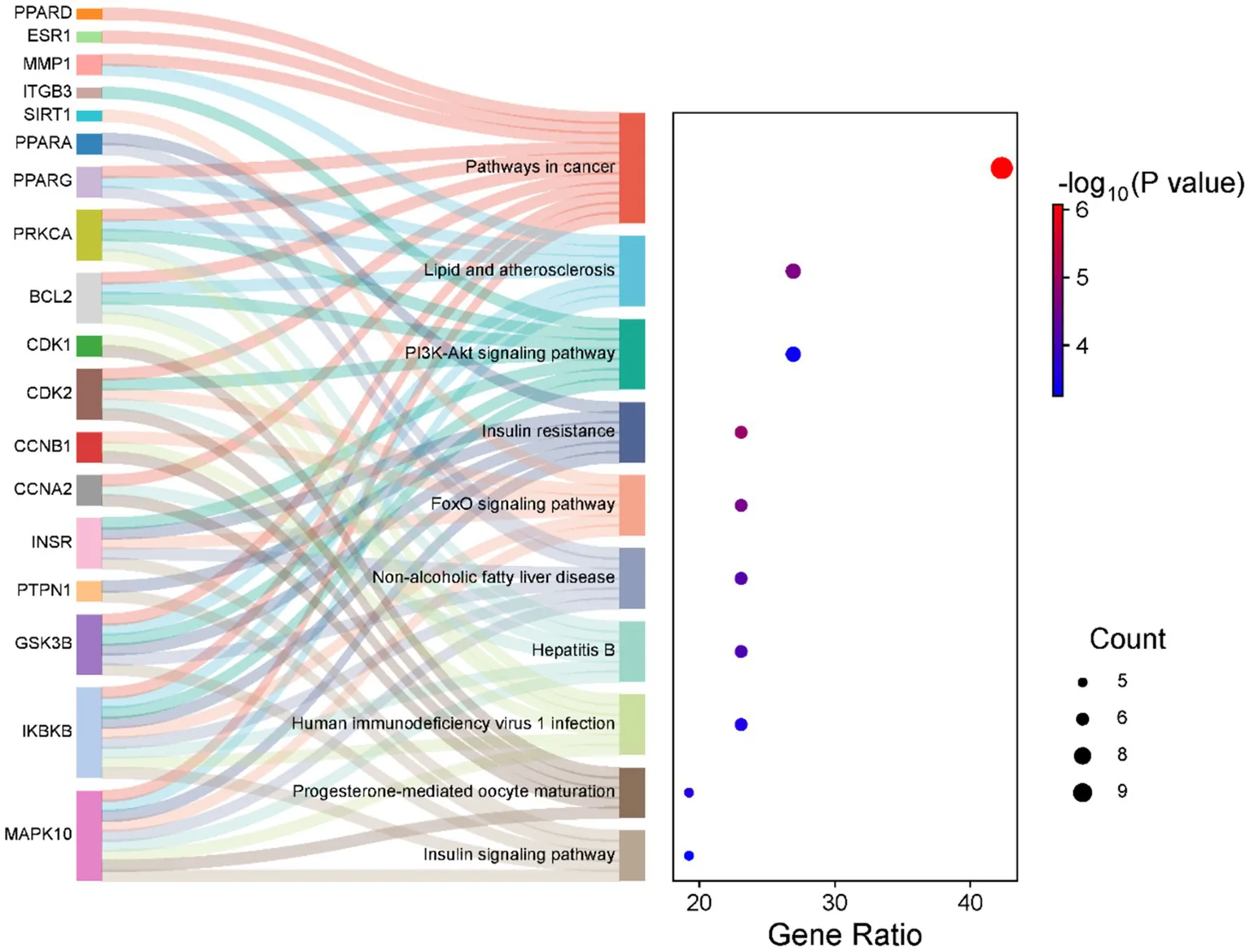
Kyoto encyclopedia of genes and genomes (KEGG) pathway enrichment analysis of the intersection targets.

KEGG pathway enrichment analysis of the intersection targets identified 68 associated signaling pathways. Using a significance threshold of *p <* 0.05 and ranking by gene ratio, the top 10 most significantly enriched KEGG signaling pathways are displayed in Figure 8. These include Pathways in cancer, Lipid and atherosclerosis, the PI3K-Akt signaling pathway, Insulin resistance, the FoxO signaling pathway, Non-alcoholic fatty liver disease (NAFLD), Hepatitis B, Human immunodeficiency virus 1 infection, Progesterone-mediated oocyte maturation, and the Insulin signaling pathway.

#### 3.3.7 Molecular docking

**Figure 9:**
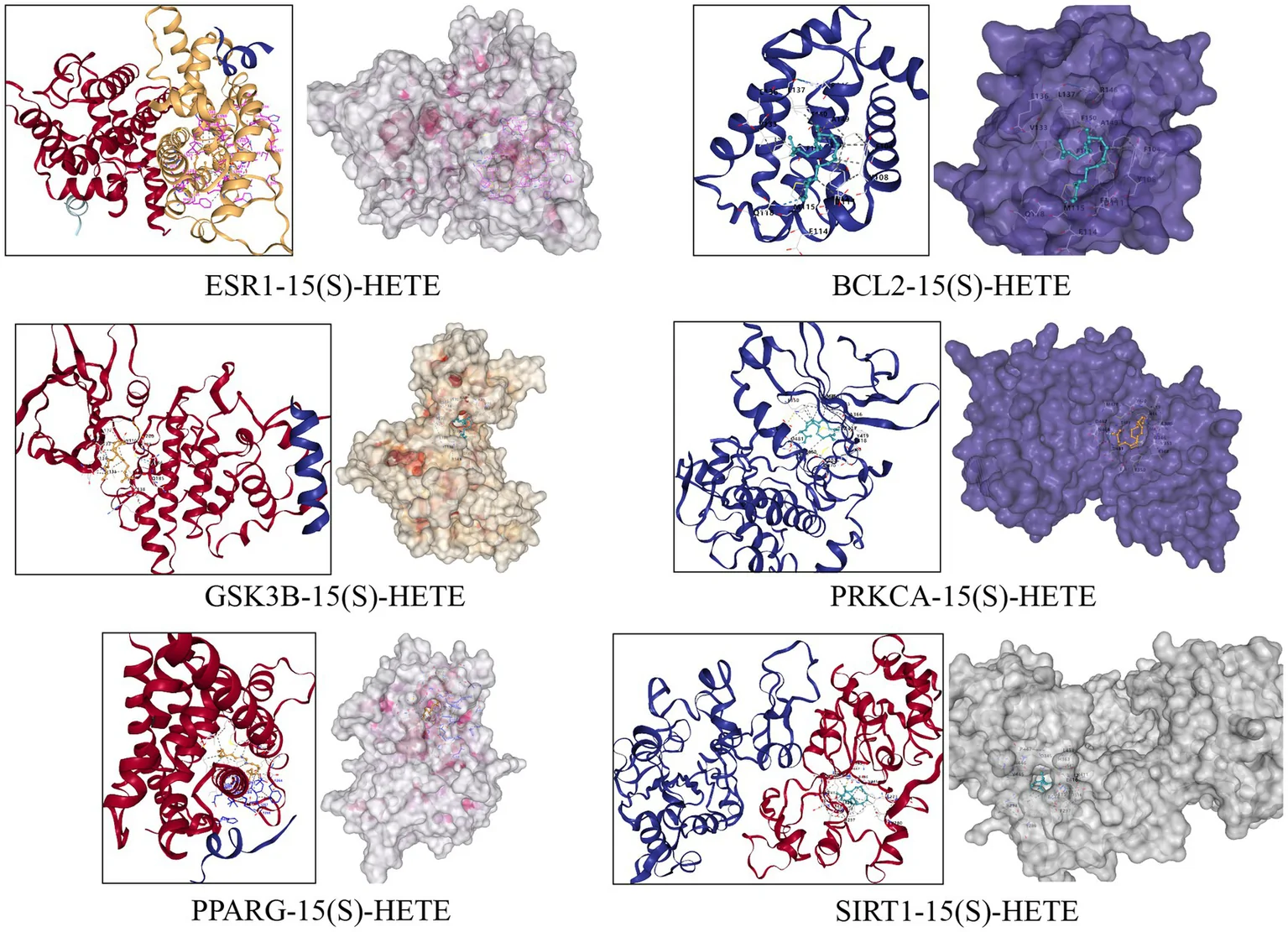
Heatmap of docking binding energies between 15(S)-HETE and core target molecules.

Conduct receptor-ligand docking experiments between 15(S)-HETE and six core targets. First, prepare the receptor protein files by importing the screened core targets into the RSCB PDB database^13^ to identify the optimal protein conformation and save it as a PDB file. Next, prepare the small molecule ligand files. Retrieve the 3D structure of 15(S)-HETE from the PubChem database, then use OpenBabel 3.1.1 to convert the 3D structure’s SDF file into MOL2 format. Import both receptor and ligand files into CB-dock2^14^ for molecular docking. Obtain the minimum binding energy value from the docking results for heatmap analysis, and visualise the docking outcomes. For details, see Table 8 and Figure 9.

**Table 8:** Binding energy and active site for molecular docking of active ingredients and core target molecules.

Active ingredients	Core targets	Binding energy (kcal/mol)	Active site
15(S)-HETE	BCL2_6GL8	−6.0	PHE104 TYR108 ASP111 PHE112 GLU114 MET115 GLN118 LEU119 VAL133 VAL134 GLU136 LEU137 GLY145 ARG146 VAL148 ALA149 PHE150 GLU152 PHE153
15(S)-HETE	GSK3B_7SXJ	−5.6	ILE62 GLY63 PHE67 GLY68 VAL69 VAL70 TYR71 GLN72 ALA83 ILE84 LYS85 GLU97 MET101 VAL110 LEU130 LEU132 ASP133 TYR134 VAL135 PRO136 GLU137 THR138 TYR140 ARG141 GLN185 ASN186 LEU188 CYS199 ASP200 PHE201
15(S)-HETE	PPARG_9F7W	−5.9	GLU259 ILE262 LYS263 PHE264 HIS266 ILE267 THR268 PRO269 GLN271 GLN273 LYS275 ILE279 ARG280 ILE281 PHE282 GLN283 GLY284 CYS285 GLN286 PHE287 ARG288 SER289 VAL290 GLU291 ALA292 GLN294 ILE326 TYR327 MET329 LEU330 LEU333 VAL339 LEU340 ILE341 SER342 MET348 LEU353 PHE363 MET364 LYS367 HIS449 MET463 SER464 LEU465 HIS466 PRO467 GLN470
15(S)-HETE	PRKCA_8U37	−6.0	LEU345 GLY346 LYS347 PHE350 VAL353 ALA366 LYS368 GLU387 THR401 GLN402 MET417 GLU418 TYR419 VAL420 ASN421 ASP424 ASN463 ASP467 ASN468 VAL469 MET470 ALA480 ASP481 PHE482 GLY483
15(S)-HETE	ESR1_7NFB	−7.5	GLU323 PRO324 PRO325 ILE326 LEU327 TYR328 MET343 LEU346 THR347 LEU349 ALA350 ASP351 GLU353 HIS356 MET357 TRP383 LEU384 ILE386 LEU387 MET388 ILE389 GLY390 LEU391 TRP393 ARG394 SER395 MET396 ASP397 HIS398 LEU403 PHE404 PRO406 MET421 ILE424 PHE425 LEU428 LEU440 ARG442 PHE445 LYS449 LYS520 GLY521 MET522 HIS524 LEU525 MET528 LEU540
15(S)-HETE	SIRT1_4I5I	−8.7	GLY261 ALA262 SER265 GLY269 ILE270 PRO271 PHE273 ARG274 ILE279 TYR280 ARG282 GLN294 PHE297 PHE312 LYS314 GLU315 ILE316 TYR317 PRO318 GLY319 GLN320 PHE321 GLN345 ASN346 ILE347 ASP348 HIS363 GLY364 ILE411 VAL412 PHE413 PHE414 GLY415 GLU416 LEU418 GLY440 SER441 SER442 LEU443 LYS444 VAL445 ARG446 PRO447 ILE510 THR511 GLU512

## 4 Discussion

Previous studies have established that AA plays a crucial role in the pathogenesis of PCOS (13, 15, 16). Although our earlier preclinical research indicated that XNYSD could modulate fatty acid metabolism through the AA pathway to improve clinical endpoints, leading to the preliminary identification of several potential molecular pathways and targets (11), a fine-grained analysis focusing on key metabolites within the AA pathway was lacking. The present study, therefore, integrated targeted metabolomics with network pharmacology to trace a path from metabolic products to core targets, delineate potential mechanisms through pathway enrichment, and ultimately link these findings to clinical outcomes. This approach investigates the therapeutic targets and associated mechanisms of XNYSD and its active ingredients in patients with phlegm-dampness type PCOS, providing a solid experimental and theoretical basis for its precise clinical application.

Baseline analysis showed that LH levels were higher in the PCOS group than in the TCM group (*p <* 0.05). Although all participants met the diagnostic criteria for PCOS prior to allocation and were comparable at enrollment, slight differences in baseline LH and BMI were observed between the two groups. This phenomenon can be reasonably explained by the intrinsic biological heterogeneity of PCOS. PCOS is not a uniform endocrine disorder but rather a spectrum encompassing distinct phenotypes that differ in gonadotropin secretion patterns, metabolic status, and body composition. Even when inclusion criteria are identical, the distribution of these phenotypes within study groups remains subject to random variation. Consequently, LH-dominant phenotypes with higher GnRH pulse frequency and preferential LH secretion may be numerically enriched in PCOS group.

Through a comparative analysis of AA-related metabolites in the follicular fluid of the PCOS and Normal groups, we found that the concentrations of 15(S)-HETE, 9(S)-HODE, PGF2α, and PGD2 were significantly increased in patients with phlegm-dampness type PCOS.

15(S)-HETE, a lipid oxidation product generated from the action of 15-lipoxygenase (15-LOX) on arachidonic acid, is recognized as a key mediator of inflammatory responses in various chronic diseases. In our study, the level of 15(S)-HETE in the follicular fluid of patients with phlegm-dampness type PCOS was significantly higher than that in the control group (*p <* 0.01), suggesting its potential role in the dysregulation of the local follicular microenvironment. Research has shown that 15(S)-HETE can enhance the local inflammatory microenvironment by activating inflammatory signaling pathways such as NF-κB to induce the release of pro-inflammatory cytokines (22), which may in turn interfere with granulosa cell function and follicular development. Furthermore, 15(S)-HETE has been confirmed to promote the expression of vascular endothelial adhesion molecules (VCAM-1, ICAM-1) and induce immune cell infiltration, thereby exacerbating the inflammatory microenvironment (23). This could potentially disrupt the stability of follicular blood supply and consequently affect oocyte maturation quality.

Previous studies have clearly indicated that 15(S)-HETE mediates endothelial cell angiogenesis by activating the PI3K/Akt/mTOR signaling pathway, highlighting its significant role in regulating cellular metabolism and signal transduction (24). Moreover, another study found that the Chinese herbal formula Yupingfeng San alleviates hepatic insulin resistance by modulating the IRS/PI3K/Akt pathway, thereby improving glucose and lipid metabolism and enhancing insulin sensitivity (25). This suggests a potential link between 15(S)-HETE and insulin resistance, where it may interfere with insulin signaling mediated by the PI3K/Akt pathway, thus acting as a bridge between metabolic disorders and reproductive dysfunction. Considering these findings, the elevated level of 15(S)-HETE in the follicular fluid of women with phlegm-dampness type PCOS may contribute to the complex pathogenesis of the condition through multiple mechanisms, including the amplification of local ovarian inflammation, interference with energy metabolism, and disruption of follicular blood perfusion. This establishes 15(S)-HETE as a crucial molecular node connecting lipid metabolism abnormalities, ovarian dysfunction, and insulin resistance.

In the present study, a significant reduction in follicular fluid 15(S)-HETE levels was observed in patients with phlegm-dampness type PCOS following administration of XNYSD. Lowering 15(S)-HETE levels may improve oocyte quality and fertilization rates by inhibiting PLA₂ activity, thereby reducing the interference of arachidonic acid metabolites within the follicular microenvironment. Indeed, clinical data have shown an inverse correlation between follicular fluid 15(S)-HETE levels and fertilization capacity, suggesting a detrimental effect on oocyte function. Consequently, modulating 15(S)-HETE concentrations could potentially optimize the fertilization microenvironment, promote the formation of two pronuclei (2PN), and thereby increase the success rate of fertilization (26). During embryo implantation, endometrial angiogenesis and cell viability are critical. Although 15(S)-HETE is known to promote angiogenesis in vascular endothelial cells by activating the PI3K/Akt/mTOR signaling pathway (24), we postulate that within the endometrium, the over-activation of this pathway could disrupt the delicate balance between proliferation and differentiation required for successful implantation. Therefore, a reduction in 15(S)-HETE levels may temper the activity of these signaling pathways, leading to a more stable endometrial vascular network and preventing premature or abnormal vascularization. This would optimize blood supply and nutritional support at the time of implantation, creating an endometrial microenvironment more conducive to embryo attachment. Furthermore, the capacity of 15(S)-HETE to induce oxidative stress [via mitochondrial and Nox4-derived reactive oxygen species (ROS)] and activate p38MAPK signaling in the vascular system suggests another potential mechanism of action (27). Given the importance of redox balance and precisely regulated MAPK signaling during implantation, reduced 15(S)-HETE levels may indirectly modulate the process of endometrial decidualization, thereby enhancing receptivity to support successful embryo implantation and early pregnancy maintenance. This could explain the observed increase in both the implantation and clinical pregnancy rates after the first embryo transfer. Research on fetal membranes has shown that 15(S)-HETE expression is upregulated during parturition, where it synergizes with inflammatory factors to activate the NF-κB/COX-2/PGE2 pathway, forming a positive feedback loop that promotes inflammatory responses associated with uterine contractions (22). Extrapolating this mechanism to the post-transfer endometrial microenvironment, a moderate reduction in 15(S)-HETE could help mitigate premature inflammatory activation, preventing the endometrium from entering a “receptive” state too early and providing a more stable environment for embryo implantation and development. Studies on bovine embryos have found that the early embryo can actively regulate 15(S)-HETE levels in the uterine microenvironment (28), indicating this molecule’s involvement in the embryo-maternal dialogue. In the context of assisted reproduction, excessively high 15(S)-HETE levels may disrupt this fine-tuned regulation, leading to imbalanced endometrial receptivity. By lowering 15(S)-HETE, a more favorable inflammatory/anti-inflammatory balance may be restored, reducing the risk of uterine contractions or endometrial dysfunction triggered by premature PGE2 elevation. Considering the pro-oxidative stress properties of 15(S)-HETE in vascular cells, its excess in the reproductive system could also impact trophoblast invasion or early placental development through ROS-mediated cellular stress. Therefore, the reduction in 15(S)-HETE could, through the aforementioned mechanisms, increase the ongoing clinical pregnancy rate following the first embryo transfer while reducing the likelihood of pregnancies failing at the biochemical stage. Firstly, reduced 15(S)-HETE mitigates oxidative damage to oocytes and granulosa cells by decreasing ROS production. Secondly, it modulates inflammatory pathways that influence follicular development and luteal function. Theoretically, these synergistic effects could improve embryo morphology and developmental potential, thereby increasing the number of transferable embryos.

9(S)-HODE, an oxidized fatty acid metabolite generated from the LOX-catalyzed metabolism of linoleic acid, is known to regulate inflammatory responses. In our study, the follicular fluid of patients with phlegm-dampness type PCOS contained significantly higher levels of 9(S)-HODE compared to the control group (*p <* 0.05). Patients with PCOS commonly exhibit a state of oxidative stress; a cross-sectional case–control study demonstrated that their serum levels of total glutathione, reduced glutathione, superoxide dismutase activity, lipid peroxidation products, and homocysteine were all significantly elevated, indicating substantial oxidative damage (29). Within the follicular fluid, high concentrations of 9(S)-HODE may not only be a product of this oxidative stress (30) but also a potent pro-inflammatory agent. By activating the G protein-coupled receptor G2A, 9(S)-HODE can trigger intracellular calcium mobilization and promote the secretion of pro-inflammatory cytokines such as IL-6, IL-8, and GM-CSF in keratinocytes (31). Furthermore, the 9(S)-HODE/GPR132 pathway plays a critical role in the reprogramming of islet-resident macrophages and subsequent islet inflammation, where it stimulates GPR132 expression and enhances the release of inflammatory factors, thereby disrupting insulin signaling and exacerbating insulin resistance (32). This aligns with the observation that, compared to non-PCOS individuals, PCOS patients have significantly elevated inflammatory markers, including white blood cell counts, CRP, TNF-*α*, IL-6, IL-18, and IL-1β, reflecting the interplay among chronic low-grade inflammation, oxidative stress, and insulin resistance (33). Clinical research has also found that oocytes that fail to form pronuclei or that degenerate after ICSI have significantly higher mean concentrations of 9-HODE and 13-HODE in their follicular fluid compared to successfully fertilized oocytes (26), suggesting that AA and linoleic acid derivatives can impair oocyte maturation and fertilization capacity. In light of the decreased normal fertilization rate in our phlegm-dampness PCOS group, we postulate that elevated levels of 9(S)-HODE in the follicular fluid serve as a key molecular bridge connecting oxidative stress, inflammation, and metabolic dysregulation in the pathogenesis of PCOS.

PGs are bioactive lipid mediators generated from arachidonic acid via the COX pathway, including PGD₂, PGE₂, PGF₂*α*, PGI₂, and TxA₂, all of which are extensively involved in female reproductive processes such as follicular development, ovulation, luteal formation and regression, and pregnancy (34–36).

Among these, PGF₂α, primarily synthesized by prostaglandin F synthase (PGFS), is considered a central regulator of luteolysis. In our study, the follicular fluid of patients with phlegm-dampness type PCOS had significantly higher levels of PGF₂α than the control group (*p <* 0.01). It is well-established that in the mammalian corpus luteum, PGF₂*α* concentration decreases during the mid-luteal phase but surges in the late-luteal phase. This surge induces luteal regression, encompassing both functional luteolysis (e.g., a sharp decline in progesterone secretion) and structural luteolysis (e.g., luteal cell apoptosis) (37, 38). Luteal function is critical for the establishment and maintenance of pregnancy, and its premature regression is closely associated with infertility and implantation failure. Patients with PCOS often have compounding factors such as hyperandrogenism, disordered GnRH pulsatility, and insulin resistance, which can interfere with granulosa cell function, inhibit luteal angiogenesis, and impair corpus luteum maturation (39). Studies have demonstrated that PGF₂*α* can induce granulosa cell apoptosis (40), further exacerbating luteal dysfunction. Moreover, the high estrogen levels often seen in PCOS patients during controlled ovarian stimulation may prematurely induce PGF₂α release by activating estrogen receptor pathways, leading to a shortened luteal phase and functional insufficiency (41, 42). Therefore, the abnormal elevation of PGF₂α is likely a significant contributor to luteal dysfunction in patients with phlegm-dampness type PCOS, consequently affecting their reproductive outcomes.

While research on the role of PGD₂ in female reproduction is less extensive, available evidence suggests its involvement in luteal function regulation through multiple pathways. On one hand, PGD₂ can enhance blood flow in the utero-ovarian vascular system, thereby promoting the transport of PGF₂α to the corpus luteum and indirectly influencing its functional and structural regression (43). On the other hand, PGD₂ can also act directly on luteal cells to inhibit the expression of steroidogenic acute regulatory protein (StAR) and 3β-hydroxysteroid dehydrogenase (3β-HSD), thereby reducing progesterone synthesis and inducing functional regression. Concurrently, it can activate apoptosis by downregulating BCL2 and upregulating Caspase-3, leading to structural luteolysis (44, 45). Research has also shown that PGF₂α and PGD₂ exhibit a synergistic effect in enhancing luteolysis when co-administered (46). In the follicular fluid of patients with phlegm-dampness type PCOS, the elevated concentrations of both PGF₂α and PGD₂ may therefore synergistically impact luteal function, further highlighting their important roles in ovarian physiology.

PPI network analysis revealed that ESR1, SIRT1, GSK3B, BCL2, PPARG, and PRKCA are potential hub targets through which XNYSD modulates 15(S)-HETE to treat phlegm-dampness type PCOS. ESR1 encodes estrogen receptor alpha, which is crucial for follicular development and ovulation. Multiple SNPs (e.g., rs104893956, rs1554259481) can alter ERα structure or ligand affinity, thereby disrupting estrogen signaling and increasing susceptibility to PCOS (47). SIRT1, a NAD^+^-dependent deacetylase, is highly expressed in ovarian follicles with dynamic changes throughout development. In PCOS patients, SIRT1 plays multiple key roles in maintaining ovarian homeostasis by promoting granulosa cell proliferation, inhibiting apoptosis, clearing rROS), maintaining mitochondrial function and energy metabolism, regulating primordial follicle activation and gap junctions, and enhancing E₂ secretion (48). The activity of GSK3β, encoded by GSK3B, is significantly enhanced in the adipocytes of PCOS patients, and its aberrant activation can exacerbate insulin resistance and hyperandrogenism (49). Research has associated specific haplotypes in Black and White populations with a higher incidence of PCOS, suggesting that genetic variations in GSK3B may be a predisposing factor (50). BCL2 is an anti-apoptotic protein; in PCOS patients, the apoptotic state of cumulus cells influences the ability of oocytes to complete *in vitro* maturation and fertilization, and the expression profile of the BCL2 gene in these cells may reflect the cytoplasmic maturation status of the oocyte (51). A splice variant of PPARG is upregulated in the granulosa cells of PCOS patients and may lead to follicular arrest by altering protein function and the transcription of downstream genes involved in cell proliferation, apoptosis, and communication (52). PRKCA has been identified as a potential target for the treatment of PCOS with asparagus, whose active components may alleviate the condition through antioxidant and anti-inflammatory effects (53).

GO and KEGG enrichment analyses indicated that XNYSD may exert its therapeutic effects on phlegm-dampness type PCOS by modulating protein phosphorylation, serine/threonine kinase activity, and pathways related to cancer, PI3K-Akt signaling, and lipid and atherosclerosis. ERα signaling drives cell proliferation in 70–80% of breast cancers, with ESR1 mutations and MAPK pathway activation considered major mechanisms of resistance to estrogen therapy (54). While 5(S)-HETE can activate the TAK1–MAP2K6–p38 MAPK pathway to promote cancer cell adhesion and invasion (55), inhibiting p38α phosphorylation not only blocks its downstream pro-mitotic signals but also reduces ERα activation, thereby suppressing ER^+^ tumor growth (56). This inhibition also thwarts endocrine resistance driven by ERα expression loss via ROS/CaMKIV and c-Src/PKC/MAPK cascades (57, 58). This suggests that the formula may indirectly regulate ERα signaling by inhibiting the 15(S)-HETE-mediated p38 MAPK and ROS/CaMKIV pathways, potentially reducing PCOS-associated tumor susceptibility. Furthermore, 15(S)-HETE can activate PPARγ/C/EBP and Akt signaling to promote preadipocyte differentiation and lipid accumulation, and it enhances adipose tissue angiogenesis by upregulating VEGF via the PI3K/Akt/mTOR pathway (59, 60). It also relies on XO/NADPH to generate ROS, which activates Syk/Pyk2/STAT1 to induce CD36 expression and OxLDL uptake, thereby promoting foam cell formation and exacerbating atherosclerosis (58). The positive correlation between serum IL-18 levels and carotid intima-media thickness (IMT) further suggests that chronic inflammation increases cardiovascular risk in this population (61). This evidence indicates that the formula may improve lipid metabolism disorders, alleviate chronic inflammation, and potentially lower cardiovascular risk by inhibiting the aforementioned PPARγ/Akt/VEGF, ROS/Syk/STAT1, and inflammatory pathways, thus providing a molecular basis for the comprehensive treatment of phlegm-dampness type PCOS.

A widely accepted principle is that a binding energy < 0 kcal·mol^−1^ indicates a spontaneous interaction between the ligand and the target, and that lower binding energies correspond to more stable ligand–receptor conformations and stronger interaction potential (62). In this study, molecular docking results showed that the binding energies of the six target proteins with the active components ranged from −8.7 to −5.6 kcal·mol^−1^, with an average of −6.62 kcal·mol^−1^, suggesting favorable binding activity across all targets. These findings indicate that the active constituents of XNYSD form stable interactions with the target proteins, supporting their potential therapeutic role in treating PCOS-related infertility.

This study has several limitations. The clinical component was a prospective non-randomized controlled trial, rendering it susceptible to selection bias and potential confounding factors (e.g., baseline patient characteristics, preferences in treatment selection). The sample size was relatively small, and both the intervention and follow-up periods were short, which limits the ability to adequately evaluate the long-term efficacy and safety of the Chinese herbal medicine. Furthermore, the outcome analysis was restricted to the first embryo transfer and did not include critical endpoints such as live birth rate, which is a primary outcome of interest in *in vitro* fertilization. In the experimental component, the metabolomic analysis did not cover the full spectrum of relevant amino acid metabolites and was confined to a preliminary investigation in follicular fluid, lacking validation in animal or cellular models. The network pharmacology approach remained exploratory, with no experimental verification of the predicted targets and pathways. Future studies should adopt a prospective, randomized, blinded design with a larger sample size and longer follow-up, incorporate more comprehensive clinical endpoints, and integrate *in vivo* and in vitro experiments for deeper mechanistic investigation.

In conclusion, this study integrates clinical outcomes, targeted metabolomics and exploratory network pharmacology to provide evidence for the therapeutic effect of XNYSD. The clinical improvements were accompanied by reduced 15(S)-HETE levels. Based on these findings, we propose a hypothetical mechanistic model: XNYSD may act through multi-component synergy to modulate targets associated with 15(S)-HETE-related pathways such as PI3K/Akt, p38 MAPK, and ROS/Syk/STAT1. This action inhibits local follicular chronic inflammation, optimizes lipid and energy metabolism, and ultimately improves IVF-ET outcomes in patients with phlegm-dampness type PCOS. These findings provide a solid theoretical basis for the application of XNYSD in the field of TCM-assisted reproduction. It must be emphasized that this mechanistic narrative remains speculative, requiring experimental validation in cellular or animal models. Our work establishes clinical and metabolomic associations, while the network pharmacology provides a hypothesis-generating framework for future mechanistic studies of XNYSD. Future research should leverage the UHPLC–MS data to further screen and identify its key active ingredients, combined with animal and cell models to thoroughly elucidate the molecular mechanisms, thereby promoting the development and clinical translation of novel drug candidates.

## Data Availability

The raw data supporting the conclusions of this article will be made available by the authors, without undue reservation.

## Footnotes

1
https://tcmsp-e.com/

2
https://herb.ac.cn/

3
https://www.genecards.org/

4
https://omim.org/

5
https://www.disgenet.org/

6
https://www.uniprot.org/

7
https://pubchem.ncbi.nlm.nih.gov/

8
http://swisstargetprediction.ch/

9
https://bioinfogp.cnb.csic.es/tools/venny/

10
https://string-db.org/

11
https://david.ncifcrf.gov/

12
https://www.bioinformatics.com.cn/

13
https://www.rcsb.org/

14
https://cadd.labshare.cn/cb-dock2/
