# Correction: Effect of Xiaonang Yusi decoction (消囊育嗣汤) on IVF outcomes in patients with phlegm-dampness type PCOS: a prospective cohort study with supporting metabolomics, network pharmacology, and molecular docking analysis

**DOI:** 10.3389/fmed.2026.1967238

**Published:** 2026-09-24

**Authors:** Xin Hu, Qingmei Jin, Hengbing Li, Jingyan Song, Haining Yuan, Ying Xu, Kailiang Ai, Zizhen Guo, Zhenni Mu, Zhengao Sun

**Affiliations:** 1 The First Clinical Medical College, Shandong University of Traditional Chinese Medicine, Jinan, China; 2 Department of Reproduction and Genetics, The Affiliated Hospital of Shandong University of Traditional Chinese Medicine, Jinan, China

**Keywords:** arachidonic acid, *in vitro* fertilization-embryo transfer, metabolomics, network pharmacology, phlegm-dampness syndrome, polycystic ovary syndrome, Xiaonang Yusi decoction

An incorrect **Funding** statement was provided. The correct funder is the Joint TCM Science & Technology Projects of National Demonstration Zones for Comprehensive TCM Reform (no. GZY-KJS-SD-2023-039); the China Postdoctoral Science Foundation (no.2024M761883) and the Taishan Scholars Program for Young Experts of Shandong Province (no. tsqn202312375).

The funder National Natural Science Foundation of China, 8247152238 to Zhengao Sun was erroneously included. The funder National Key Research and Development Program of China, 2024YFC3505800 to Zhengao Sun was erroneously included. The funder Natural Science Foundation of Shandong Province, ZR2022LZY009 to Zizhen Guo was erroneously included.

The original version of this article has been updated.

