# Supplementary material for: Effect of Xiaonang Yusi decoction (消囊育嗣汤) on IVF outcomes in patients with phlegm-dampness type PCOS: a prospective cohort study with supporting metabolomics, network pharmacology, and molecular docking analysis
Source: Front Med (Lausanne). 2026 Feb 10;13:1680327. doi: 10.3389/fmed.2026.1680327 (PMC12929475; doi:10.3389/fmed.2026.1680327)

| Metabolite Name | TCM | PCOS | Normal | *P* |
| --- | --- | --- | --- | --- |
| Arachidonic acid | 382.2810(234.3537, 486.6231) | 286.1308(237.3738, 336.7273) | 284.0735(208.0631, 426.8479) | 0.064 |
| Docosahexaenoic acid | 286.6820(186.1826, 347.5149) | 226.9457(188.2338  ,262.4421) | 300.5251(190.6525,304.8691) | 0.140 |
| 12(S)-HETE | 5.5440(3.5504, 14.0339) | 7.6071(5.4317,11.9893) | 7.1025(3.5024, 8.7585) | 0.605 |
| 15(S)-HETE | 13.9761(11.3715, 15.8558)^#^ | 15.4561(12.5770, 18.2398)* | 13.4157(11.2487, 16.2974) | 0.023 |
| 9(S)-HODE | 7.7536(6.0197, 12.4736) | 8.4930(6.7959, 10.3107)* | 6.1164(4.8714, 8.2102) | 0.046 |
| 13(S)-HODE | 13.6302(10.4899, 16.2930) | 14.5976(11.5304, 18.3795) | 14.2348(11.7348, 16.0935) | 0.624 |
| Prostaglandin D2 | 6.8192(3.5953, 20.1305)* | 11.2241(2.9368, 22.1797)* | 3.4359(1.8585, 9.0110) | 0.044 |
| Prostaglandin F2α | 2.0650(1.0046  , 5.9754) | 3.4160(1.2798, 9.6134)* | 2.0650(1.0046, 5.9754) | 0.023 |
| Thromboxane B2 | 1.8631(1.5535, 3.8815) | 2.5286(1.6844, 3.2227) | 1.3549(1.1340, 2.2881) | 0.059 |

Note: *Indicates a statistically significant difference compared with the Normal group; ^#^Indicates a statistically significant difference compared with the PCOS group;


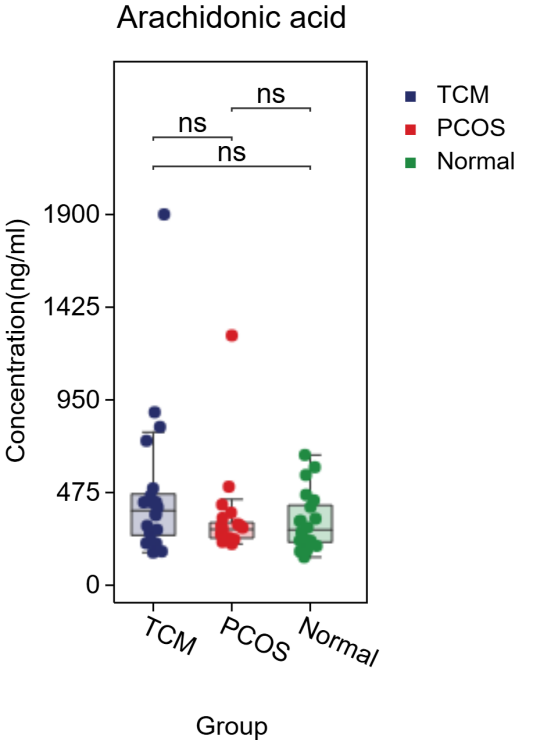

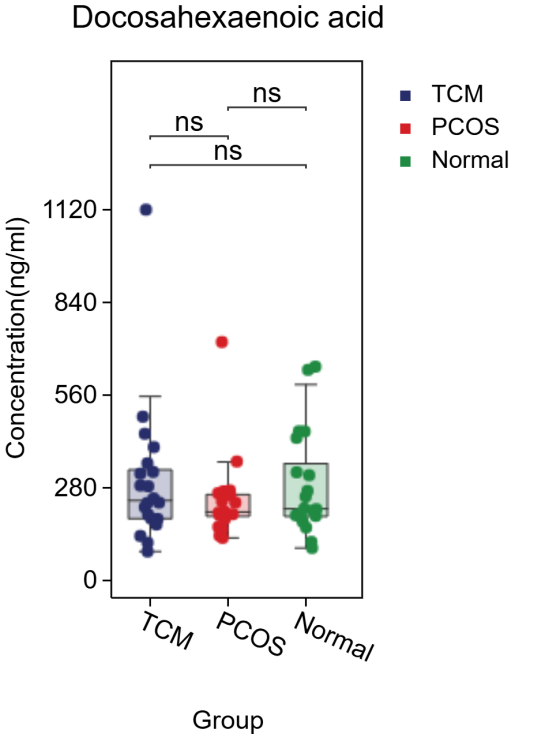


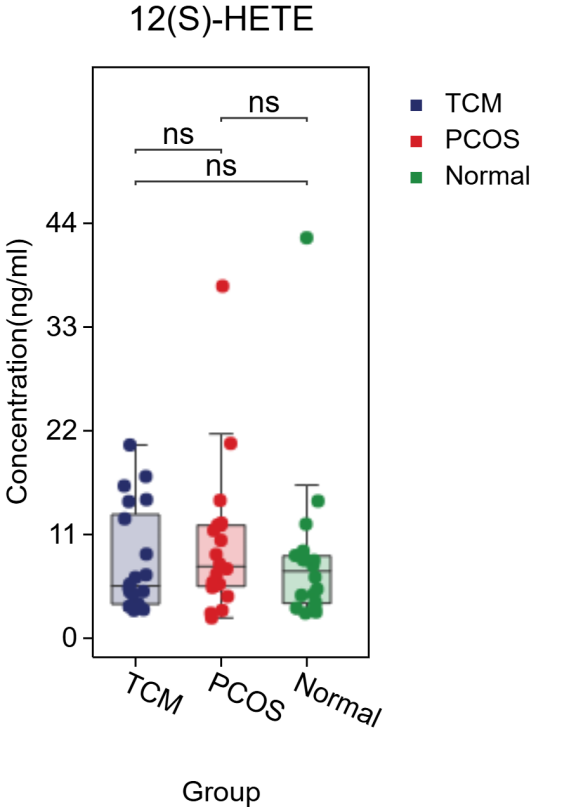

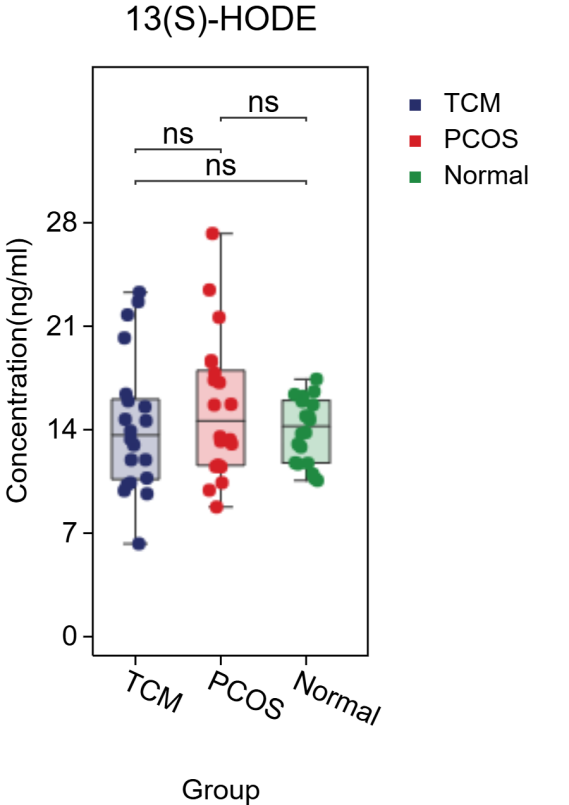


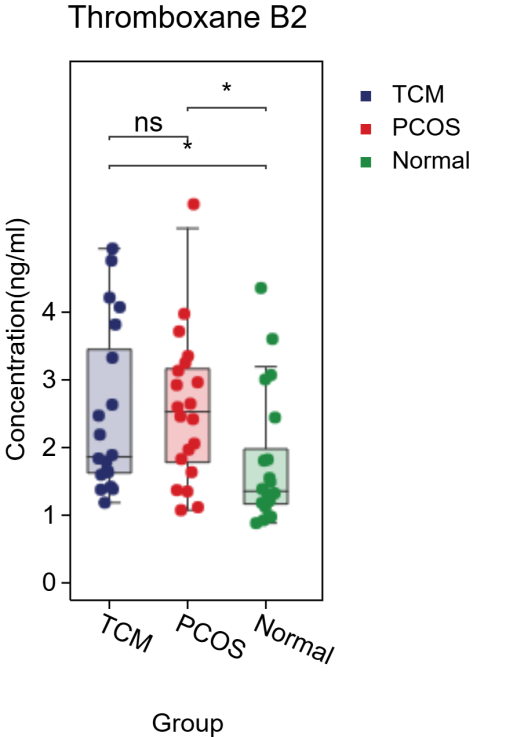

Supplement: Supplementary file 1 [file Data_Sheet_1.docx]
