# Supplementary figures and images for: Effect of Xiaonang Yusi decoction (消囊育嗣汤) on IVF outcomes in patients with phlegm-dampness type PCOS: a prospective cohort study with supporting metabolomics, network pharmacology, and molecular docking analysis
Source: Front Med (Lausanne). 2026 Feb 10;13:1680327. doi: 10.3389/fmed.2026.1680327 (PMC12929475; doi:10.3389/fmed.2026.1680327)

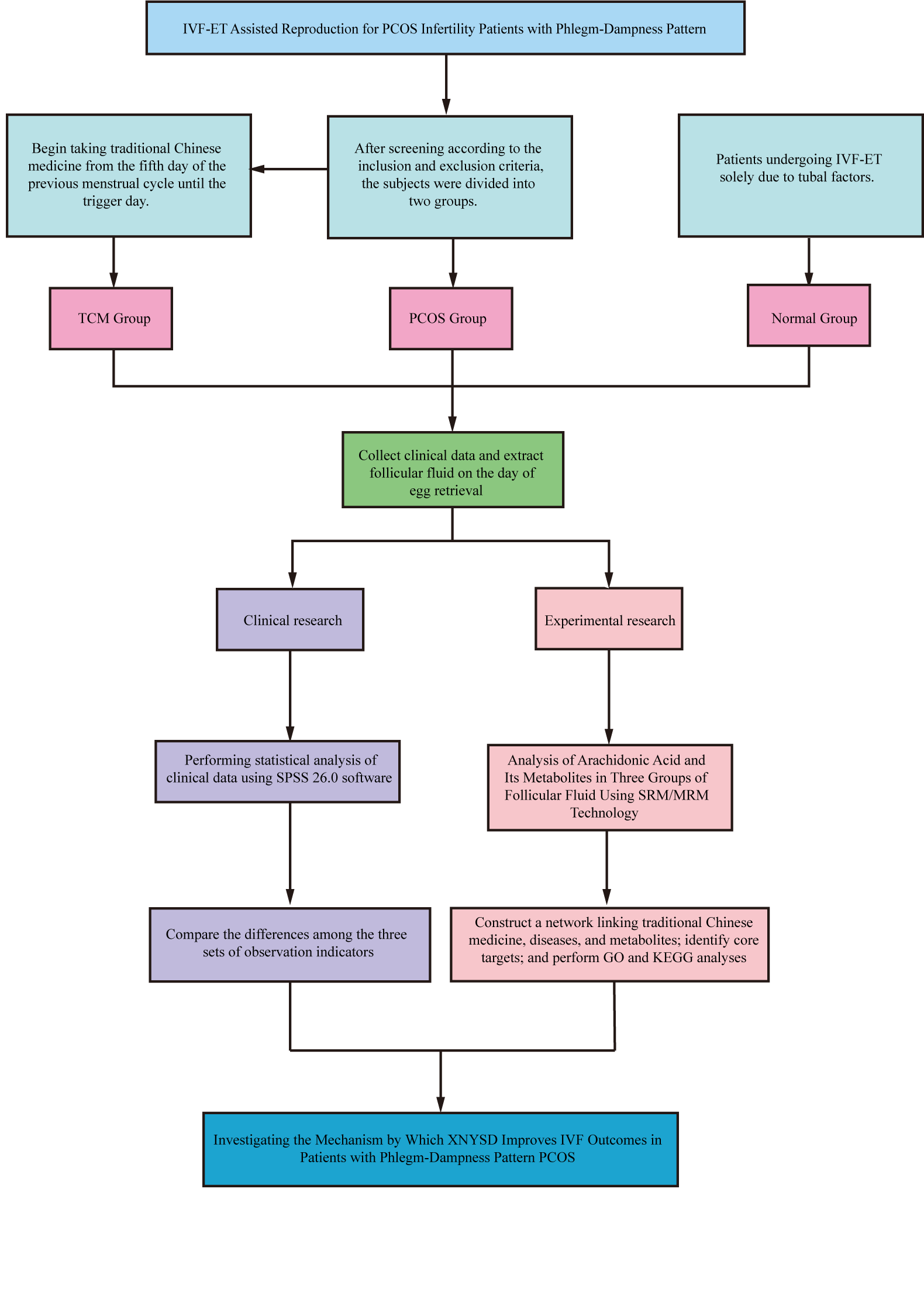

Supplement: Supplementary file 2 [file Image_1.TIF]
